# Nanoparticles containing intracellular proteins modulate neutrophil functional and phenotypic heterogeneity

**DOI:** 10.3389/fimmu.2024.1494400

**Published:** 2025-01-22

**Authors:** Leonore Raudszus, Farbod Bahreini, Susanne Allan, Kai-Uwe Kalies, Charles C. Caldwell, Kathrin Kalies

**Affiliations:** ^1^ Institute of Anatomy, University of Luebeck, Luebeck, Germany; ^2^ Institute of Biology, University of Luebeck, Luebeck, Germany; ^3^ Department of Surgery, College of Medicine, University of Cincinnati, Cincinnati, OH, United States

**Keywords:** neutrophils, heterogeneity, intracellular content, cell-derived nanoparticles, damage-associated molecular pattern, resolution

## Abstract

Neutrophils are rapidly recruited to sites of infection, injury, or to immune complexes. Upon arrival, they initiate degranulation, release reactive oxygen species (ROS), and/or nuclear extracellular traps (NETs) to eliminate invading microorganisms, clear debris, or remove abnormal immunoglobulins. While these processes ideally trigger healing and a return to balance, overshooting neutrophil function can lead to life-threatening infections such as sepsis or persistent inflammation observed in various autoimmune diseases. However, recent evidence highlights a phenotypic and functional heterogeneity of neutrophils that extends well beyond their traditional - potentially harmful- role as first responders. For example, neutrophils regulate ongoing inflammation by modulating macrophage function through efferocytosis, T cell responses by antigen presentation and the release of cytokines. The factors that induce neutrophil differentiation into activating or regulatory phenotypes remain poorly defined. Here, we hypothesize that intracellular components that have been released into the extracellular space could contribute to the phenotypic heterogeneity of neutrophils. To find out, we used nanoparticles composed of intracellular proteins (cell-derived nanoparticles, CDNPs) and analyzed their effects on cultured murine bone marrow neutrophils (BMN). We observed that CDNPs activate BMN transiently with an increase in the expression of CD11b without triggering classical effector functions. Additionally, CDNPs induce the secretion of IL-10, shift PMA-induced cell death toward apoptosis, and increase the expression of CD80. Mechanistically, our findings indicate that 26% of BMNs ingest CDNPs. These BMNs preferentially express CD54+, fail to migrate toward CXCL12, exhibit diminished responses to LPS, and undergo apoptosis. These data identify CDNPs as biomaterials that modulate neutrophil behavior by fine-tuning the expression of CD11b and CD80.

## Introduction

Neutrophils are among the first cells to infiltrate sites of infection or injury, as well as locations where immune complexes are deposited in autoimmune diseases. They are capable of inducing robust responses, which can potentially cause collateral tissue damage (1). Conventionally, neutrophils have been described as short-lived cells with a defined set of effector functions such as phagocytosis, degranulation, ROS release, formation of NETs, and the secretion of specific cytokines. However, recent studies have uncovered unexpected phenotypic heterogeneity and functional plasticity in neutrophils, suggesting that these cells can significantly influence the duration, severity, and outcome of subsequent immune responses (2). Thus, neutrophils may either exacerbate ongoing tissue-damaging inflammation or promote regulatory, resolving, and tissue-repairing processes (3, 4). The factors that regulate this neutrophilic heterogeneity are poorly defined. Their identification will be important to develop new therapeutic approaches.

This study explores the hypothesis that the differentiation of neutrophils into either activating or regulatory phenotypes may be influenced by proteins that are typically localized intracellularly but are released during accidental cell injury. The presence of such intracellular proteins in the extracellular space has traditionally been associated with the initiation of inflammation, even in the absence of pathogens. These intracellular components are termed “Danger-” or “Damage-Associated Molecular Patterns” (DAMPs) because they are recognized by Pattern Recognition Receptors, thereby activating innate immune cells and inducing inflammation (5, 6). Typical examples include HMGB1, histones, S100 proteins, and heat shock proteins, which have been studied extensively *in vitro* and *in vivo*, where each protein was introduced individually for investigation (7, 8). However, the complexity of *in vivo* scenarios is far greater, involving a diverse array of intracellular proteins, such as cytoskeletal elements, chaperones, and enzymes, as well as combinations of these proteins with nucleic acids and lipids, released either individually or as complexes. Currently, there is no data available on the impact of these protein mixtures on neutrophils. To address this, we employed CDNPs, which predominantly consist of intracellular proteins and are structured as nanoparticles ranging from 100 to 200 nm in size. The primary proteins present in CDNPs include Annexins (ANXA1-5), HSP60, actin, galectin, and vimentin, among others, as well as small amounts of nucleic acids (9, 10).

According to our current understanding of how intracellular contents influence innate immune cells, CDNPs would be categorized as DAMPs. However, previous studies have shown that CDNPs exert regulating capacities. Thus, CDNPs accelerate and improve the healing of antibody-induced skin wounds in the mouse model for the skin blistering disease epidermolysis bullosa acquisita (9). CDNPs also showed promise in critical medical situations such as sepsis, as observed in the murine cecal ligation and puncture model, where they positively influenced the host response (10).

In this study, we assessed the effects of CDNPs on various neutrophil functions, including phagocytosis, ROS release, NETosis, cytokine secretion, apoptosis, migration, and the expression of phenotypic markers such as CD11b, CD80, CD86, MHCII, and CD54 in cultures of murine bone marrow cells (BMC). We found that CDNPs did not activate traditional effector functions; however, a transient increase in CD11b, upregulation of CD80 expression and apoptosis were observed, indicating that intracellular content indeed seems to regulate inflammatory processes by modulating the phenotype of neutrophils. Further research is needed to decipher and better understand the effects of intracellular content on the heterogeneity of neutrophil subsets.

## Materials and methods

### Mice/bone marrow cells

For all experiments, BMC were isolated from inbred C57BL/6J mice aged 8-12 weeks. Mice were anesthetized with CO_2_ and killed by cervical dislocation. Tibiae and femora were flushed with HBSS (Thermo Fisher Scientific, USA). For each experiment, the bone marrow of two mice was combined. The BMC were flushed through a cell strainer, pelleted, and red blood cells were lysed with Ammonium-Chloride-Potassium buffer. Then, the BMC were resuspended in RPMI 1640 (Lonza, Switzerland), supplemented with 5% FBS, 1 x Non-essential amino acids, 1 mM Sodium Pyruvate, 100 U/ml Penicillin/100 µg/ml Streptomycin, 7.5 mg/L Gentamycin, 5.25 µg/L 2-mercaptoethanol, 2 mM L-Glutamine (all Thermo Fisher Scientific, USA), 20 mM Hepes Sodium Salt (Sigma Aldrich (Germany). Cells were counted and adjusted to 2 x 10^6^ cells/mL. 10^6^ BMC in 0.5 mL medium were used per sample unless indicated otherwise. All murine experiments were approved by the Institutional Animal Care and Use Committee of the University of Cincinnati (protocol no. 10-05-10-01). The approval date was June 26, 2018. Detailed materials are provided in the STAR*Method format in the supplement.

### CDNP isolation and preparation

CDNPs were isolated from the murine fibroblast cell line MC3T3E1 (Deutsche Sammlung von Mikroorganismen und Zellkulturen, Braunschweig, Germany). The fibroblasts were disrupted by ultrasound, and the fragments were pelleted at 5000 g for 30 min. The supernatant was ultracentrifuged at 50000 g for 150 min to pellet the particles. To dissolve the remaining membranes, chloroform (C. Roth GmbH & Co. KG, Germany) was added at a final concentration of 5% for 15 seconds. Phase separation was achieved by centrifugation at 9000 g for 15 min. In the supernatant remained the CDNPs. To ensure a consistent quality of the protein pattern, each CDNP preparation was analyzed by SDS Page Gels (pre-cast 4-12% Bis-Tris Midi Protein Gels, Thermofisher Scientific, USA). To compensate for batch variations, several CDNP preparations were combined into pools. Endotoxin was removed by treatment with 1% Triton X-114 (Sigma Aldrich, Germany) as described in (11). PBS that was used for control samples was also treated with Triton X-114. Endotoxin levels were measured with a Limulus Amebocyte assay using the commercially available Pierce™ Chromogenic Endotoxin Quant Kit (Thermo Fisher Scientific, USA) according to the manufacturer’s instructions. 0.1 EU/mL was used as a lower limit for endotoxin contamination.

### CFSE-labeling of CDNPs

The CDNPs were labeled with Carboxyfluorescein succinimidyl ester (CFSE) as previously described (10). The CDNP suspension was incubated with 10 µM CFSE (Becton Dickinson, USA) per 100 µg/mL CDNPs at 37°C for 20 min. Excessive CFSE was captured by adding a medium containing 10% fetal bovine serum (Thermo Fisher Scientific, USA) and incubation for 10 min. The PBS control sample was treated the same way.

### Intracellular and surface labeling of cells using flow cytometry

For intracellular and surface labeling of cells, the samples were centrifuged and treated with Fc-receptor blocking buffer (containing anti-mouse CD16/32 antibody and 5% rat serum (Thermo Fisher Scientific, USA)) for 10 min, followed by incubation with antibodies for 20 min. BMC were washed and analyzed with the Attune^®^ NxT™ Acoustic Focusing Cytometer (Thermo Fisher Scientific, USA) or the LSR II Flow Cytometer (Becton Dickinson, USA). The following fluorescent-labeled antibodies were used: Anti-Histone H3 (citrulline R2 + R8 + R17) Primary Antibody (Polyclonal), FITC Anti-Myeloperoxidase antibody (clone: 2D4) (both from Abcam, UK); APC anti-mouse CD86 Antibody (clone: GL-1), APC-Cy7 anti-mouse CD86 Antibody (clone: GL-1), Brilliant Violet 605™ antimouse/human CD11b Antibody (clone: M1/70), Pacific Blue™ anti-mouse I-Ab Antibody (MHC II) (clone: AF6-120.1), Pacific Blue™ anti-mouse Ly-6G Antibody (clone: 1A8), PE anti-mouse CD80 Antibody (clone: 16-10A1), PerCP/Cy5.5 anti-mouse Ly-6G Antibody (clone: 1A8), APC anti-mouse Ly-6G Antibody (clone: 1A8), Brilliant Violet 421™ antimouse/human CD11b Antibody (clone: M1/70) (all from BioLegend, USA); Anti-mouse CD16/32 Antibody (clone: 93) (from BioLegend, USA or Thermo Fisher Scientific, USA); APC Hamster Anti-Mouse CD54 (clone: 3E2), APC Rat Anti-CD11b (clone: M1/70), APC-Cy7 Rat Anti-CD11b (clone: M1/70), FITC Hamster Anti-Mouse CD54 (clone: 3E2), Fixable Viability Stain 570, PE Hamster Anti-Mouse CD54 (clone: 3E2), PE Rat Anti-Mouse Ly-6G (clone: 1A8) (all from Becton Dickinson, USA), PE CD11b Monoclonal Antibody (clone: M1/70), Fixable Viability Dye eFluor 780; Alexa Fluor 700 Goat anti-Rabbit IgG (H+L) Cross-Adsorbed Secondary Antibody [all from Thermo Fisher Scientific (USA)].

### Functional assays

Unless indicated otherwise, the cells were always pretreated with 10 µg/mL CDNPs for 45 min before further stimulation or direct analysis. For some experiments, the cells were stimulated with 100 ng/mL LPS (Lipopolysaccharides from Escherichia coli O111:B4, Sigma Aldrich, Germany) for 24 hours at 37°C between preincubation and further stimulation or direct analysis.

### Chemotaxis

BMC were isolated and pretreated as described above, transferred to 5 mL tubes and counted; 10^6^ cells were added to the top well insert of a Transwell^®^ plate (Corning, USA). The bottom well contained 100 ng/mL Recombinant Mouse CXCL12 (SDF-1α) (BioLegend, USA). After 3 hours of incubation at 37°C, non-migrated cells from the top well insert and migrated cells from the bottom well were transferred to 5 mL tubes, respectively, pelleted, and counted before flow cytometry analysis. The percentage of neutrophils in the top and bottom wells, respectively, was determined, and the percentage of migrated neutrophils was calculated.

### Phagocytosis

*E. coli* (K-12 strain, BioParticles™, Alexa Fluor™ 488 conjugate, Thermo Fisher Scientific, USA) were prepared according to the manufacturer’s instructions. The particles conjugated to a fluorescent dye and opsonized with *E. coli*-specific polyclonal IgG antibodies (*E.coli* Opsonizing Reagent, Thermo Fisher Scientific, USA) were incubated with pretreated BMC for 15 min at 37°C in a water bath. The cells were fixed by adding 500 μL of 1% paraformaldehyde (Thermo Fisher Scientific, USA) washed and stained for flow cytometry. Ly6G+/CD11b+ granulocytes emitting a fluorescence around 520 nm were considered as neutrophils that had phagocytosed *E. coli* particles. To discriminate internalized *E. coli* BioParticles™ from those bound to the cell surface, the samples were reanalyzed after quenching extracellular fluorescence with trypan blue (Sigma Aldrich, Germany) (final concentration 0.43 mg/mL; **Supplementary Figure 1**). Additionally, a second phagocytosis assay was performed using pHrodo™ dye, which emits fluorescence only in the acidic milieu inside the phagolysosome. The *E. coli* particles (pHrodo™ Red *E. coli* Bioparticles™ conjugate, Thermo Fisher Scientific, USA) were prepared according to the manufacturer’s instructions. The opsonized and conjugated particles were incubated with pretreated BMC (for 60 min at 37°C). Then, the cells were fixed with 1% PFA for 5 min, washed, and stained with antibodies as described above. Ly6G+/CD11b+ granulocytes emitting a fluorescence around 585 nm were considered as neutrophils that had phagocytosed particles.

### ROS release

BMC were isolated and pretreated as described above. The cells were loaded with 2 µM Dihydrorhodamine 123 (DHR 123) (Sigma Aldrich, Germany) for 10 min. The cells were put on ice to stop the reaction and washed twice before antibody staining. The cells were kept on ice during the whole staining procedure. Ly6G+/CD11b+ granulocytes were analyzed for their green fluorescence (Mean Fluorescence Intensity, MFI of DHR 123).

### NETosis assay

BMC were isolated and pretreated as described. They were then exposed to 100 ng/mL Phorbol 12-myristate 13-acetate (PMA, Sigma Aldrich, Germany) for 3 hours at 37°C. After washing and fixing in 1% PFA for 5 min, the cells were resuspended in blocking buffer and stained with a primary H3 antibody for 30 min at room temperature. Following another wash, cells were incubated with Alexa Fluor 700-conjugated antibodies against H3, FITC-conjugated anti-MPO antibodies, surface markers Ly6G, and CD11b for 30 min, then analyzed by flow cytometry. Ly6G+/CD11b+/H3+/MPO+ cells were identified as NETosing cells.

### Apoptosis assay

BMC were isolated and pretreated as described above. The cells were stimulated with 100 ng/mL PMA (Sigma Aldrich, Germany) for 3 hours at 37°C. The cells were pelleted, washed, and first stained with anti-Ly6G and anti-CD11b antibodies. Then, the cells were stained with a Fixable Viability Stain (FVS) according to the manufacturer’s instructions. Then, active caspase 3 was stained according to the manufacturer’s instructions (FITC Active Caspase-3 Apoptosis Kit, Becton Dickinson, USA). Cells were identified as follows: viable (FVS-/casp3-), apoptotic (FVS+/casp3+), necrotic (FVS+/casp3-) cells.

### IL-10 and TNF-α release

BMC were isolated and pretreated as described above and stimulated with 100 ng/mL LPS for 24 hours at 37°C. The supernatants were analyzed for IL-10 and TNF-α levels using a Cytometric Bead Array Kit (Mouse Inflammation Kit, Becton Dickinson) according to the manufacturer’s instructions.

### Statistical analysis

Data were analyzed using GraphPad Prism Version 5.03 (GraphPad Software Inc., USA). Linear regressions for standard curves were fit with Microsoft Excel 365 (Microsoft, USA). Data in scatter or bar plots are expressed as mean ± standard deviation (SD). Non-parametric tests (Mann-Whitney and Kruskal-Wallis test) were used for experiments with one factor. Experiments with two factors were compared with one- or two-way ANOVA with Dunn’s or Bonferroni posttests. To account for multiple comparisons, as is the case in chemotaxis and phagocytosis assays, we report the adjusted significance level when one dataset was compared to multiple others (12). We considered test results with a p-value < 0.05 statistically significant. Statistical analysis focused on the differences between PBS- and CDNP-treated BMN. **Figure 5** compares CDNP^-^ and CDNP^+^ BMN. All significant and nonsignificant changes are shown in the figure’s graphs. We used OpenAI’s ChatGPT (version as of December 2024) for its assistance in language editing.

## Results

### Bone marrow neutrophils recognize CDNPs but exhibit only transient activation

**Figure 1:**
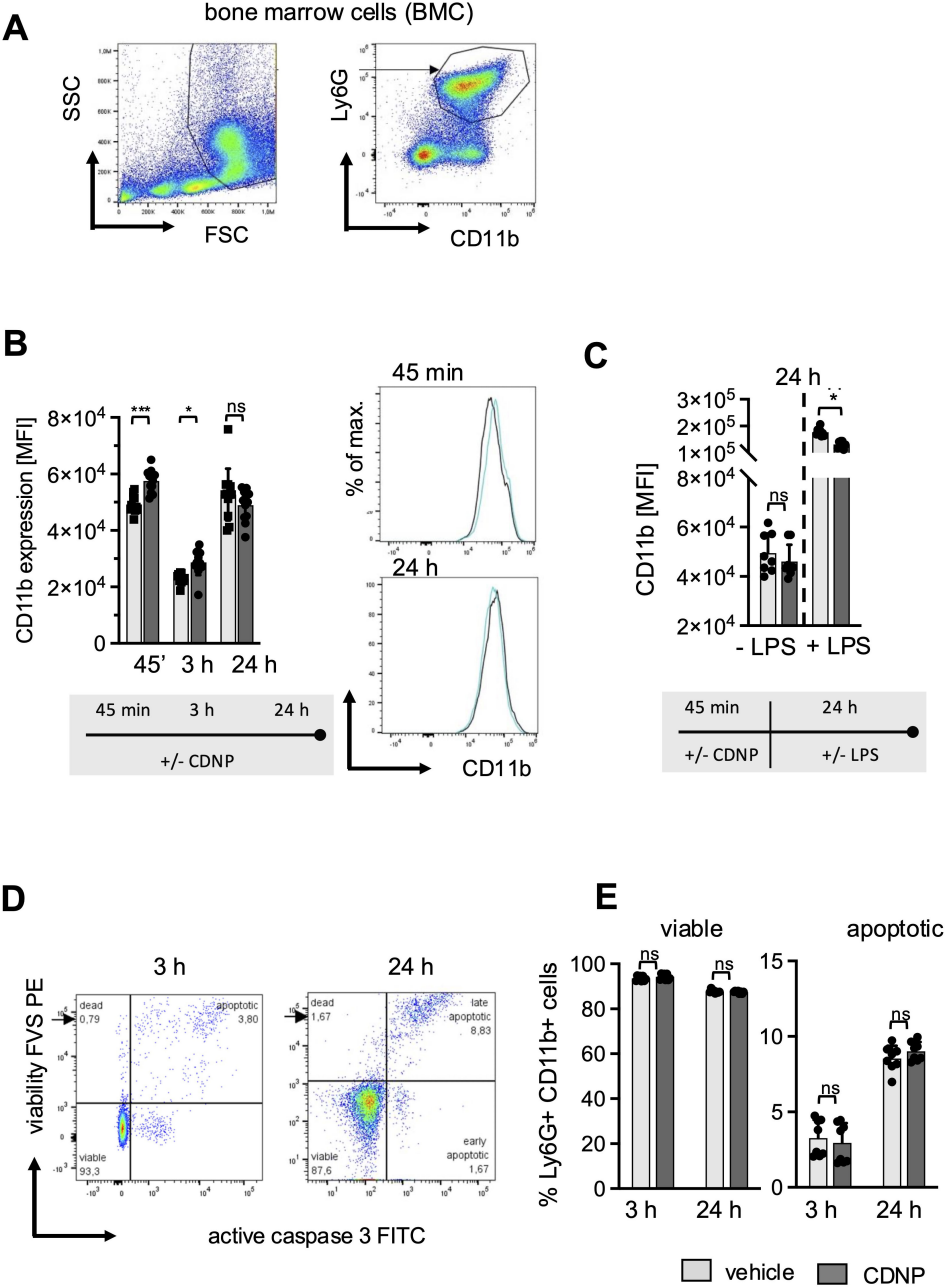
CDNPs activate Ly6G+CD11b+ BMN rapidly and transiently without affecting cell viability. BMC from C57BL6 mice were incubated with 10 µg/mL CDNPs and analyzed at indicated time points. **(A)** BMN were identified as Ly6G+CD11b+ cells by flow cytometry as depicted. **(B)** The MFI of CD11b in BMN was measured 45 min, 3 hours, or 24 hours after incubation with CDNPs. Representative histograms are shown for 45 min and 24 hours (right). **(C)** The MFI of CD11b was measured in BMN after preincubation with 10 µg/mL CDNPs for 45 min following addition of 100 ng/mL LPS and culture for 24 hours. **(D)** The percentage of viable and apoptotic Ly6G^+^CD11b^+^ BMN was determined by staining with Fixable Viability Stain (FVS) and an anti-active caspase-3 antibody, 3 hours and 24 hours after incubation. A representative dot plot is shown (viable: FVS⁻Casp3⁻; apoptotic: FVS^+^Casp3^+^). **(E)** Data obtained in **(D)** are expressed as mean ± SD. Data from 2 independent experiments with n = 8-12 (2 x 4-6 pseudo-replicates) are expressed as mean ± SD. Significance was determined with two-way ANOVA and Bonferroni post-tests. * p < 0.05, *** p < 0.001. Bars: PBS (pale gray), CDNP (dark gray)). The incubation periods are illustrated below the graphs.

CD11b, an established early activation marker for neutrophils, has been shown to have increased expression in response to CDNPs during ongoing inflammation in sepsis (10). To find out whether CDNPs would impact CD11b expression under steady-state conditions, we cultured BMC from naïve C57BL/6 mice and exposed them to CDNPs for 45 min, 3 hours, and 24 hours. Neutrophils were identified by their expression of CD11b and Ly6G (referred to as BMN, **Figure 1A**). The results depicted in **Figure 1B** reveal that even though CD11b is constitutively expressed on BMN, CDNPs increase its expression rapidly 45 min after starting the culture. This augmented CD11b expression persisted for 3 hours but ceased after 24 hours. Notably, both groups exhibited a transient decrease in CD11b expression after 3 hours of culturing. This phenomenon resolved after 24 hours of culture and might be attributed to the adaptation process to the culture flasks. To mimic inflammatory conditions LPS was added to the cultures for 24 hours after a 45 min preincubation period with either CDNPs or vehicle. A 3.5-fold increased expression of CD11b was found in the control group, which was lowered significantly by the presence of CDNPs (**Figure 1C**). Finally, we determined whether CDNPs would affect the viability of BMN during the 3 hours and 24 hours culture period. BMN were stained with Fixable Viability Stain (FVS) and caspase 3 to assess the percentage of viable and apoptotic cells. The percentage of apoptotic cells increased by 8.5% in the PBS group and slightly higher (9%) in the CDNP group within 24 hours but was not significantly different between the two groups (**Figures 1D, E**). In summary, the initial upregulation of CD11b expression indicates that BMN recognize CDNPs. This recognition leads to a transient activation and is followed by a diminished responsiveness to LPS at later time points without compromising the cell viability.

### CDNPs do not induce classical effector functions in BMN but stimulate the secretion of IL-10

**Figure 2:**
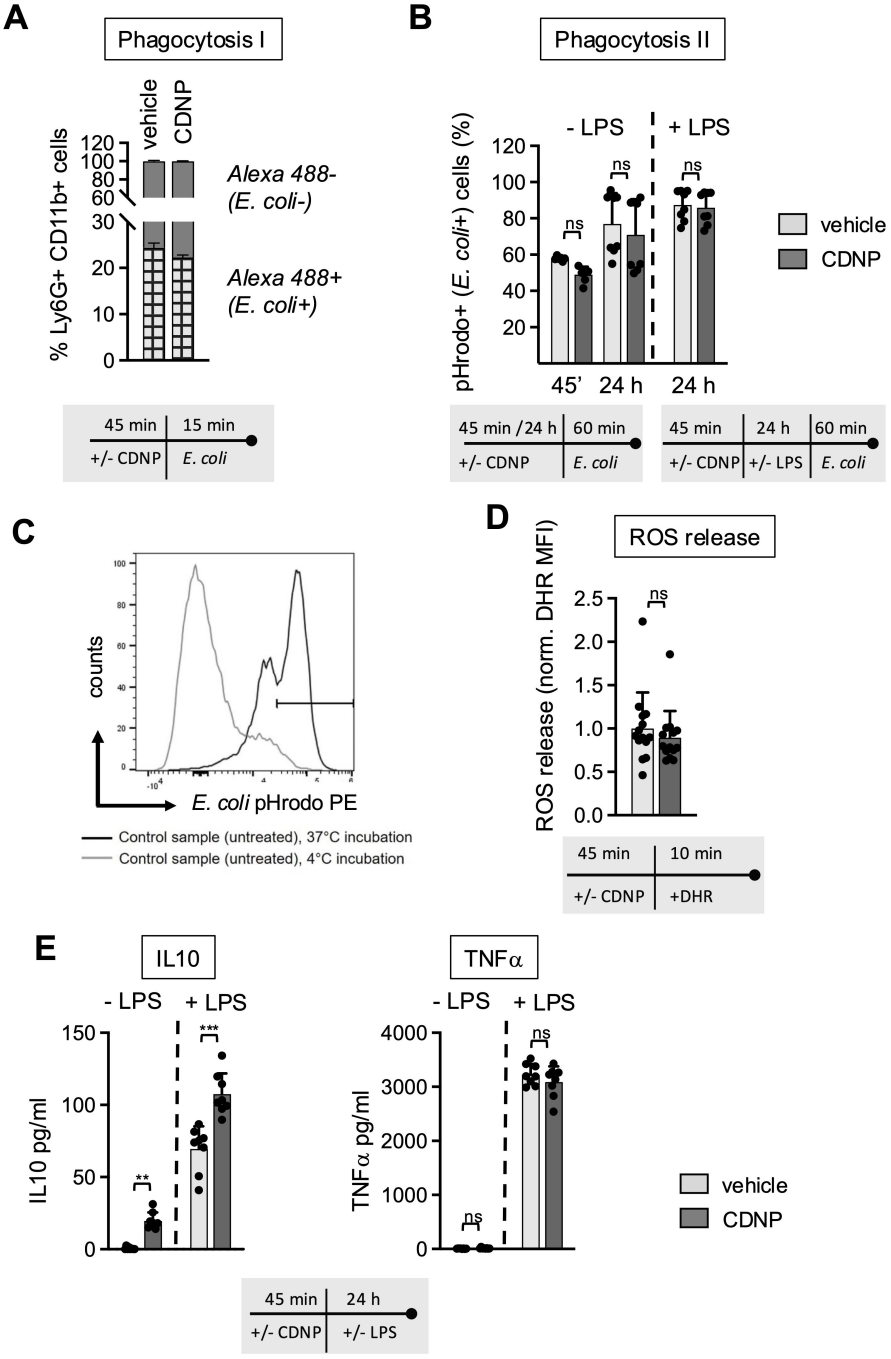
CDNPs do not induce antimicrobial effector functions; instead, they support the secretion of IL-10. **(A–E)** BMC from C57BL6 mice were preincubated with 10 µg/mL CDNPs for 45 min and subsequently treated as described. **(A)** Opsonized, Alexa Fluor 488-positive *E*. *coli* particles were added to CDNP-preincubated BMC and incubated for an additional 15 min. The percentage of Ly6G+CD11b+Alexa Flour 488+ BMN (checked bar) was determined using flow cytometry before and after treatment with trypan blue (**Supplementary Figure 1**). **(B)** To exclude surface-bound *E*. *coli* from analysis, CDNP-preincubated BMC were exposed directly to pHrodo^TM^ Red *E*. *coli* Bioparticles^TM^ conjugates for 60 min without LPS (left bars) or cultured for an additional 24 hours with addition of 100 ng/mL LPS before exposure to pHrodo^TM^ Red *E*. *coli* Bioparticles^TM^ conjugates for 60 min (right). The percentage of Ly6G+CD11b+pHrodo+ (587 nm) BMN was determined by flow cytometry. **(C)** A representative plot for incubation at 37°C and 4°C is shown. **(D)** Intracellular ROS release in Ly6G+CD11b+BMN was measured by staining CDNP-preincubated cells with DRH 123 for 10 min at 37°C. The MFI of DHR 123 was measured using flow cytometry. **(E)** BMC were incubated with 10 μg/mL CDNPs for 45 min and cultured without further stimulation or with 100 ng/mL LPS for 24 hours. IL-10 and TNF-α were analyzed in the supernatant using a Cytometric Bead Array. Data are expressed as mean ± SD, pooled data from 2 independent experiments with n = 8 (2 x 4 pseudo-replicates) per group. Data in **(D)** were pooled from 3 independent experiments with n=14 (2 x 4 and 1 x 6). Significance was determined with a two-way-ANOVA. ** p < 0.01 *** p < 0.001, adjusted significance level α(k=2) = 0.025. Bars: PBS (pale gray), CDNP (dark gray)). The incubation periods are illustrated below the graphs. ns means not significant.

The biphasic behavior of CD11b expression on BMN upon exposure to CDNPs raises the question of whether CDNPs promote further inflammatory responses in neutrophils. Considering the particulate structure and intracellular content of CDNPs, one would anticipate that CDNPs act as DAMPs and trigger antimicrobial effector functions. To explore this further, phagocytosis, ROS release, and cytokine expression were assessed in BMN subjected to a 45-minute preincubation with CDNPs. The impact of CDNPs on phagocytosis was evaluated using two complementary assays. The first method assessed the direct uptake of fluorescently labeled *E. coli* (Alexa 488) by neutrophils, providing a straightforward measure of phagocytic activity. In this assay, approximately 24% of BMN internalized *E. coli* within 15 minutes. In CDNP-treated cells, this uptake slightly decreased to 22%, though the difference was not statistically significant (**Figure 2A**; **Supplementary Figure 1**). To confirm that the observed uptake reflected true internalization rather than surface binding, the second assay used pHrodo™-labeled *E. coli*. This dye fluoresces only upon entry into the endosomal compartment, allowing a more specific assessment of bacterial ingestion. In this method, LPS was added. Over extended incubation periods (45 min and 24 hours), the percentage of BMN that internalized pHrodo™-labeled *E. coli* increased from roughly 50% to 80%. Notably, no differences were observed between CDNP-treated and control cells, even after 24 hours of LPS exposure (**Figures 2B, C**). To assess whether CDNPs induce the production of reactive oxygen species, we compared the levels of oxidized Dihydrorhodamine by measuring the intracellular Mean Fluorescence Intensity (MFI). Interestingly, no effect on ROS release was found in CDNPs-treated BMN (**Figure 2D**). Next, we assessed the secretion of the anti-inflammatory cytokine IL-10 and the pro-inflammatory cytokine TNF-α. As shown in **Figure 2E**, CDNP treatment alone stimulated the secretion of IL-10 while not affecting TNF-α levels. This pattern became more pronounced upon stimulation with LPS. A marked elevation of IL-10 secretion was found in the CDNP-treated BMC, whereas TNF-α levels remained unchanged when compared to the control group. These findings, together with the data on phagocytosis and ROS release, suggest that CDNPs do not activate BMN. Instead, CDNPs rather enhance the regulatory activities of BMN.

### CDNPs shift the PMA-induced cell death toward apoptosis

**Figure 3:**
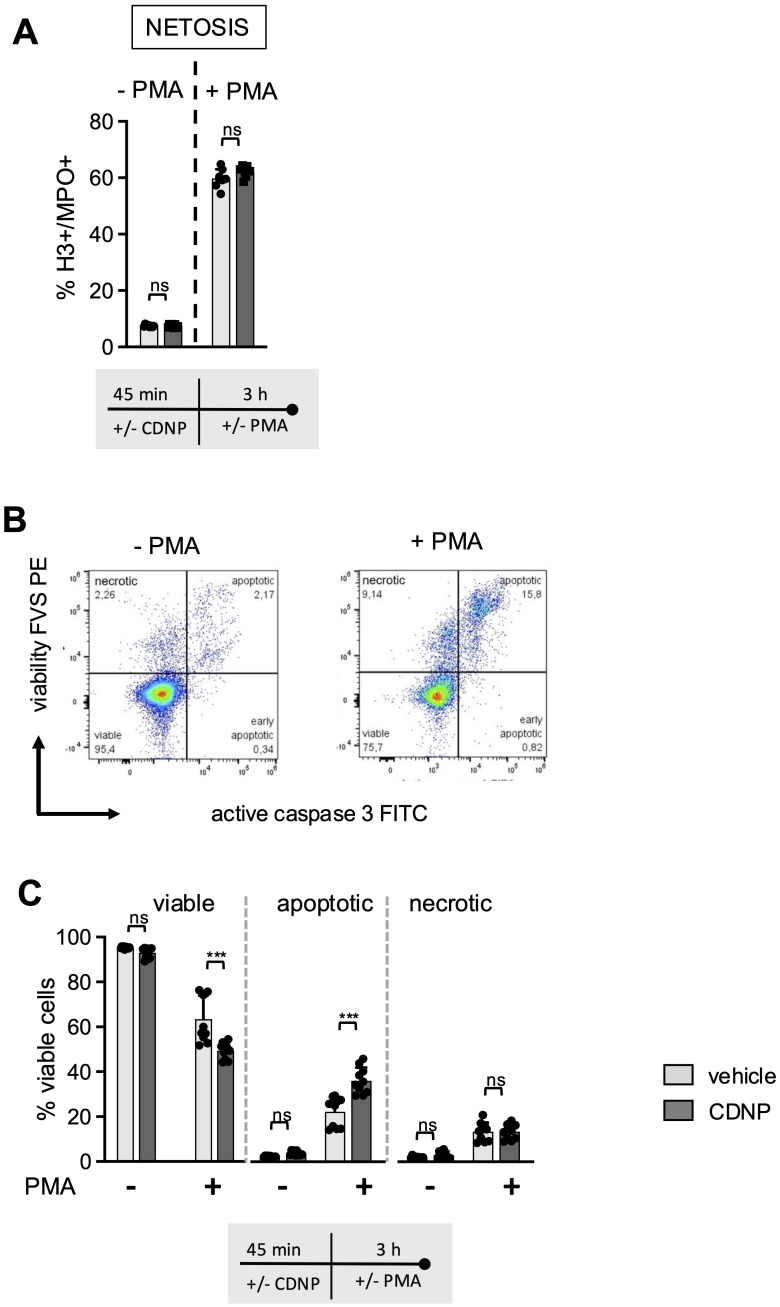
CDNPs shift PMA induced cell death in Ly6G+CD11b+BMN towards apoptosis. **(A–C)** BMC from C57BL6 mice were preincubated with 10 µg/mL CDNPs for 45 min and subsequently stimulated with 100ng/ml PMA for 3 hours. **(A)** NET formation was identified by staining with fluorescently labeled antibodies against H3 and MPO (H3+/MPO+) in Ly6G+CD11b+ BMN. **(B)** A representative dot plot of vehicle-treated BMN is shown. Cell death rates within the Ly6G+CD11b+ cell population were determined by flow cytometry using FVS and active caspase 3 (casp3) (viable: FVS-casp3-; apoptotic: FVS+casp3+; necrotic: FVS+casp3-). **(C)** Data obtained in **(B)** are expressed as mean ± SD, pooled data from 2 independent experiments with n = 10 (2 x 5 pseudo-replicates) per group. Significance was determined with a two-way ANOVA with Bonferroni posttests. *** p < 0.001. In **(A)** for the PMA treated group n = 7-8 (2 x 3-4 pseudo-replicates) are shown. One pair of values was excluded In the PMA treated group because the CDNP-treated sample had apparently not been stimulated with PMA. Bars: PBS (pale gray), CDNP (dark gray). The incubation periods are illustrated below the graphs. ns means not significant.

Next, we investigated whether CDNPs would impact the mode of cell death in BMN and introduced PMA to the cultures. PMA is known to induce the release of NETs while simultaneously initiating the process of neutrophil death (13, 14). BMN were preincubated for 45 min with CDNPs and cultured for an additional 3 hours with or without the addition of PMA. As shown in **Figure 3**, PMA induced the release of NETs in approximately 60% of the BMN, as judged by Histone 3 (H3) and Myeloperoxidase (MPO) positivity. CDNPs alone did not induce NETs and did not influence the PMA-induced NET release (**Figure 3A**). To differentiate between necrotic and apoptotic BMN, a live-dead staining (FVS) and caspase 3 staining for the detection of necrotic cells and apoptotic cells, respectively, were used (**Figure 3B**). As depicted in **Figure 3C**, PMA activation induced cell death in approximately 37% of the cells. Significantly more dead BMN (51%) were found in the CDNP-treated group. However, upon comparing the incidence of apoptotic and necrotic BMN, it becomes obvious that the presence of CDNPs shifted the cell death toward apoptosis (**Figure 3C**).

### CDNPs enhance the antigen-presenting capacity of BMN by increasing the expression of CD80

**Figure 4:**
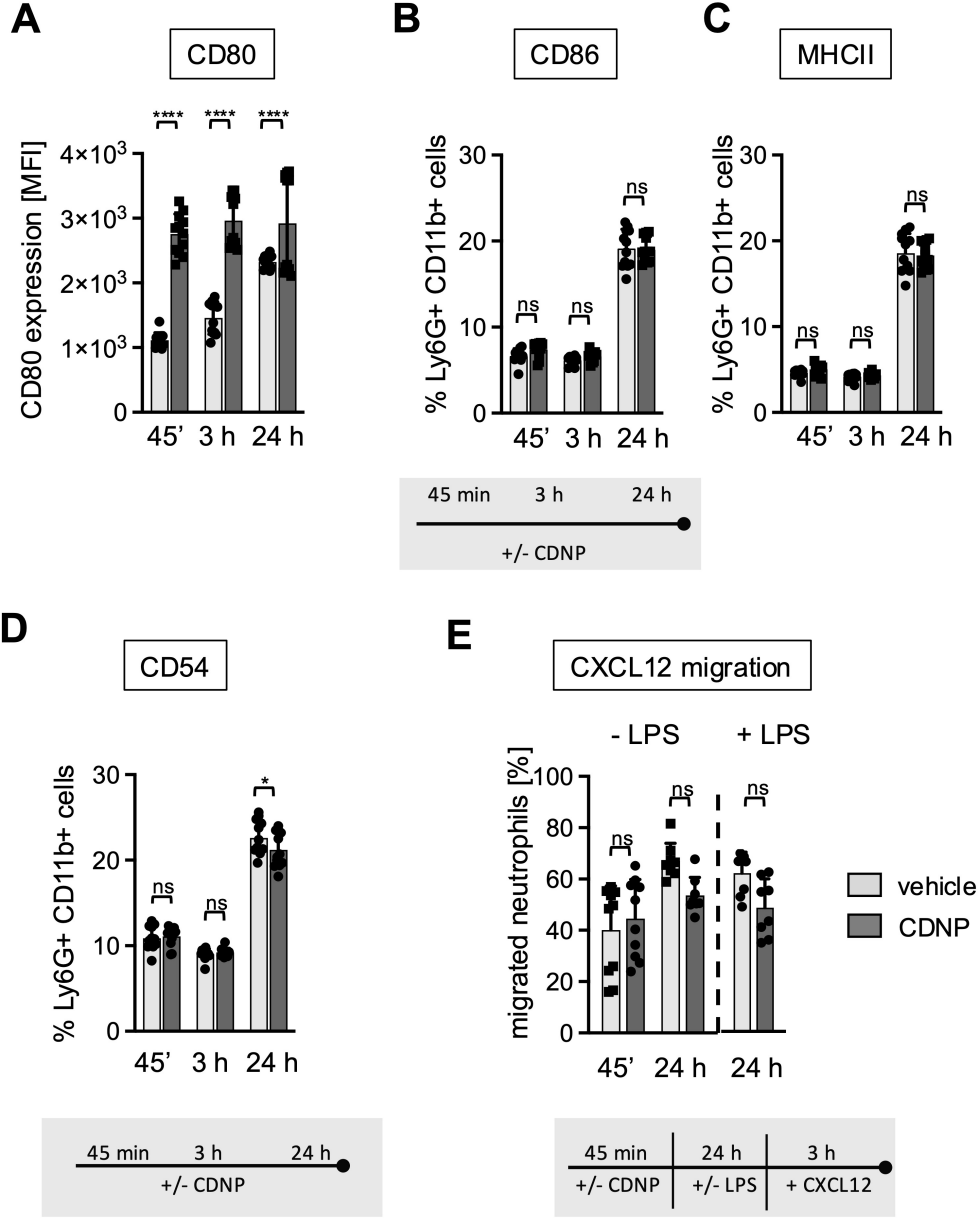
20% of BMN mature to a CD86+/MHCII+/CD54+ subset during culture. **(A–D)** BMC from C57BL6 mice were incubated with 10 µg/mL CDNPs for 45 min, 3 hours or 24 hours. **(A)** The MFI of CD80 expression and **(B–D)** The percentage of MHCII, CD86 and CD54 expressing BMN was determined by flow cytometric analysis. Representative histograms for CD80, CD86, MHCII, and CD54 are shown in **Supplementary Figure 2**. **(E)** Following exposure to CDNPs for 45 min and subsequent culture for 24 hours with or without 100 ng/mL LPS, BMC were stimulated with 100 ng/mL of CXCL12 for 3 h in transwell plates. The percentage of migrated BMN was calculated by flow cytometry. Data are expressed as mean ± SD, pooled data from 2 independent experiments with n = 8-12 (2 x 4-6 pseudo-replicates) per group. Significance was determined with a two-way ANOVA with Bonferroni post-tests. * p < 0.05, **** p < 0.0001, adjusted significance level α (k=2) = 0.025. Bars: PBS (pale gray), CDNP (dark gray). The incubation periods are illustrated below the graphs. ns means not significant.

After having established that the CDNP-induced early increase in CD11b expression on BMN is not followed by a full-scale activation but rather by a transition to a less responsive phenotype, the question arises whether other surface molecules, especially those involved in more regulatory functions, would be modulated by exposure to CDNPs. We chose CD80, CD86, and MHCII as markers for antigen presentation and the adhesion molecule CD54 (ICAM-1), primarily expressed on activated and aged neutrophils, and monitored their expression for 45 min, 3 hours, and 24 hours (4, 15–17). As shown in **Figure 4**, CD80, constitutively expressed on all BMN (18, 19), exhibited a 2.5-fold increase in expression after 45 min of culture with CDNPs compared to the control group. Exposure to CDNPs not only accelerated but also augmented CD80 expression during the complete culture period (**Figure 4A**). In contrast, CDNPs did not markedly affect MHC II, CD86, or CD54 expression at any time point. All three markers increased over time in the control and CDNP-treated groups from approximately 5% after 45 min to 20% after 24 hours (**Figures 4B–D**; **Supplementary Figure 2**). A slight yet significant reduction of CD54 expressing BMN was observed in the CDNP-treated group (**Figure 4D**). CD54 is known for its role in mediating adhesion during the transmigration of endothelial cells (17, 20). To investigate whether the emergence of these 20% aged CD54+ BMN would affect the migratory behavior, we conducted transmigration assays. Because CD54 expression correlates with the chemokine receptor CXCR4 we exposed BMN to CXCL12, a ligand for CXCR4 (21, 22). Following a 45-min incubation with CDNPs or vehicle, BMN were either directly exposed to CXCL12 for 3 hours or cultured for an additional 24 hours with or without LPS, then exposed to CXCL12 for 3 hours (**Figure 4E**). No significant changes could be observed. Regardless of the culture duration, 40-67% of the BMN migrated towards CXCL12, with LPS addition showing no impact. Notably, although not statistically significant, the number of migrating BMN increased slightly and CDNP-treated groups tended to migrate to a lesser extent than the controls after 24 hours of culture, irrespective of the presence of LPS. In summary, CDNPs selectively upregulated CD80 in synergy with aging while leaving other antigen presenting markers unaffected.

### CDNPs are specifically ingested by CD54+ neutrophils that do not migrate towards CXCL12

**Figure 5:**
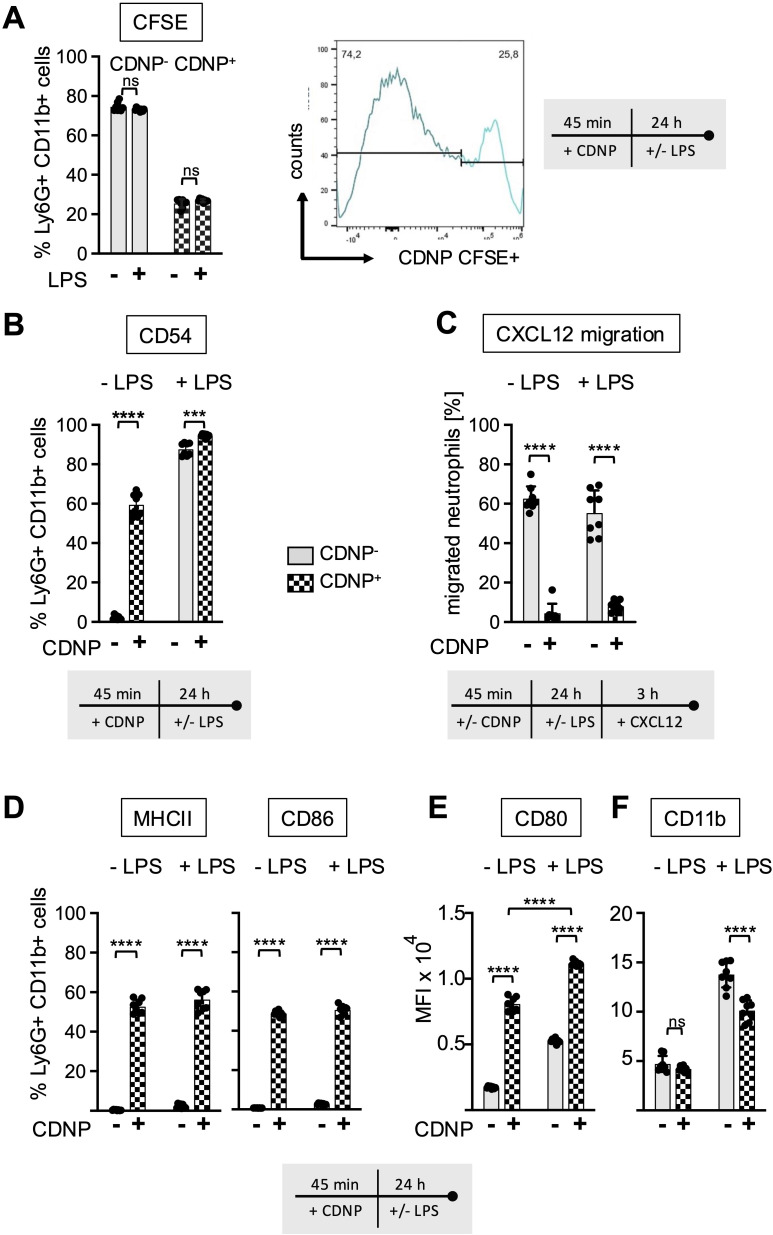
CDNPs are internalized by 26% of BMN and modulate their expression of CD80 and CD11b. BMC from C57BL6 mice were preincubated with 10 µg/mL CFSE-labeled CDNPs for 45 min and subsequently cultured with or without LPS for 24 hours **(A)** The percentage of the CFSE-labeled subset among the Ly6G+CD11b+ BMN was determined by flow cytometry. To establish that CFSE-labeled CDNPs were internalized rather than surface-bound, samples were analyzed before and after treatment with trypan blue in initial experiments (**Supplementary Figure 3**). **(B)** The percentage of CD54 expressing cells in CFSE- (CDNP^-^) and CFSE+ (CDNP^+^) BMN is shown. **(C)** Following exposure to CDNPs for 45 min and subsequent culture for 24 h with or without 100ng/mL LPS, BMC were stimulated with 100 ng/mL of CXCL12 for 3 h in transwell plates. The percentage of migrated CDNP^-^ and CDNP^+^ BMN was calculated by flow cytometry. **(D)** The percentage of CD86 and MHCII expressing cells in CDNP^-^ and CDNP^+^ BMN is shown. Representative histograms for CD80, CD86, MHCII and CD54 are shown in **Supplementary Figure 4** **. (E, F)** The MFI of CD11b **(E)** and CD80 **(F)** was assessed in CDNP^-^ and CDNP^+^ BMN. Data are presented as mean ± SD, pooled data from 2 independent experiments with n = 8-12 (2 x 4-6 pseudo-replicates) per group. Significance was determined with a two-way with Bonferroni posttests. *** p < 0.001; **** p < 0.0001. Bars: CDNP^-^ BMN (pale gray), CDNP^+^ BMN (patterned). The incubation periods are illustrated below the graphs. **Supplementary Figure 3** provides additional information on the quenching of surface-bound CFSE. The incubation periods are illustrated below the graphs or in **(A)** on the right side. ns means not significant.

**Figure 6:**
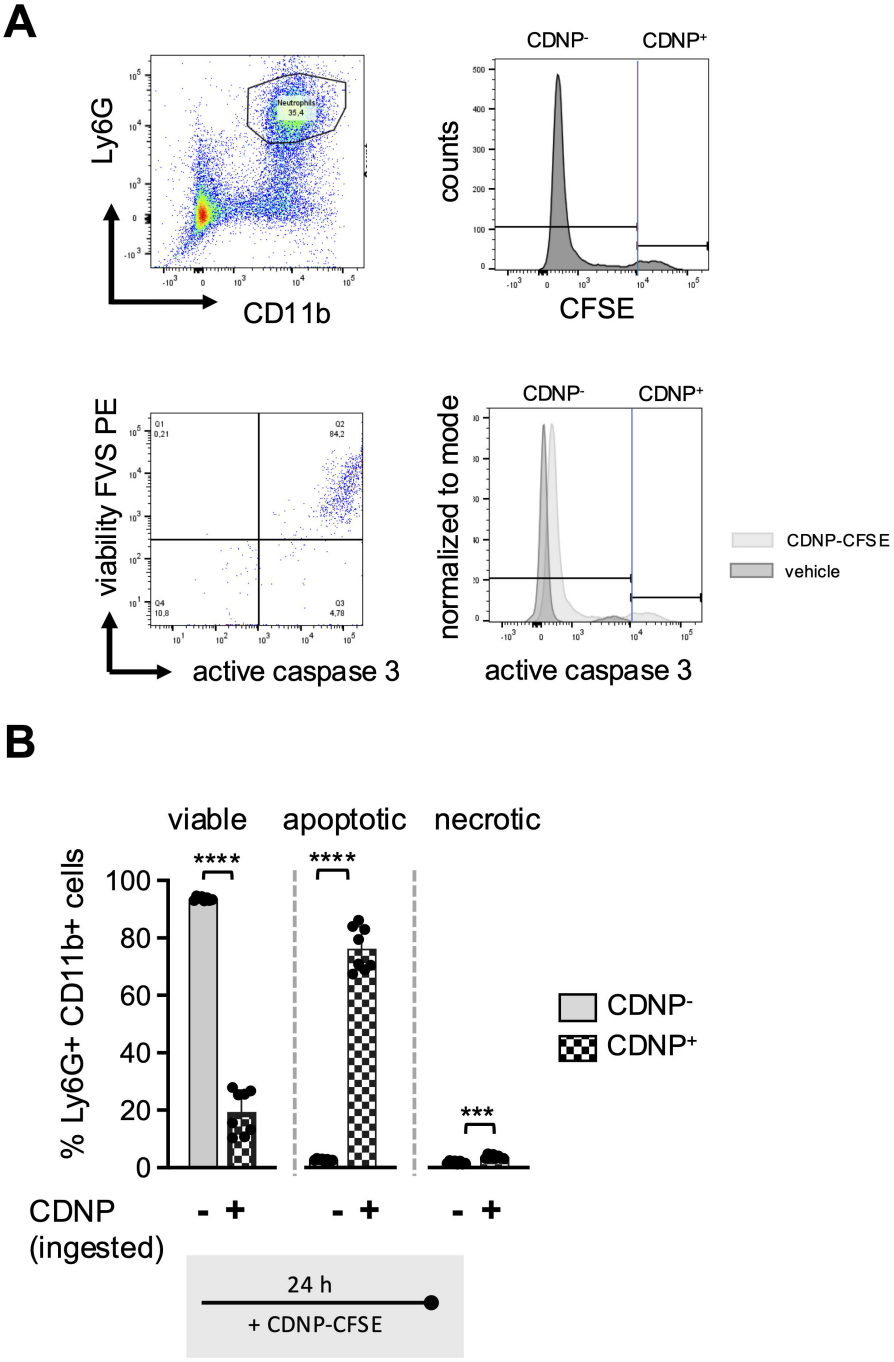
80% of the CDNP+BMN become apoptotic. BMC from C57BL6 mice were preincubated with 10 µg/mL CFSE-labeled CDNPs for 45 min and subsequently cultured for 24 hours. The percentage of the CFSE-labeled subset among the Ly6G+CD11b+ BMN was determined by flow cytometry. To establish that CFSE-labeled CDNPs were internalized rather than surface-bound, samples were analyzed before and after treatment with trypan blue in initial experiments (**Supplementary Figure 3**). **(A)** Dot plot and histograms show a representative example of the percentage of the CFSE-labeled BMN subset within the Ly6G^+^CD11b^+^ BMNs (upper panel), as well as the distribution of viable, apoptotic, and necrotic BMNs within the CDNP^-^ and CDNP^+^ Ly6G^+^CD11b^+^ cell population, identified by staining with FVS and an active caspase-3 antibody. Viable cells were defined as FVS⁻Casp3⁻, apoptotic cells as FVS^+^Casp3^+^, and necrotic cells as FVS^+^Casp3⁻ (lower panel). **(B)** The percentage of viable apoptotic and necrotic cells in CFSE- (CDNP^-^) and CFSE+ (CDNP^+^) BMN is shown. Data are presented as mean ± SD, pooled data from 2 independent experiments with n = 8-12 (2 x 4-6 pseudo-replicates) per group. Significance was determined with a Kruskal-Wallis test. *** p < 0.001; **** p < 0.0001. Bars: CDNP^-^ BMN (pale gray), CDNP^+^ BMN (patterned).

Previous reports have indicated that CD54 expression on neutrophils correlates not only with aging but also with increased phagocytic activity (16, 17). To investigate whether this also applies to the ingestion of CDNPs, we labeled CDNPs with CFSE prior to preincubation with BMC for 45 min. The cells were then cultured for an additional 24 hours, with or without LPS stimulation, and CD54 expression was assessed on CDNP^+^ and CDNP^-^ BMN. Overall, approximately 26% of BMN ingested CDNPs, indicated by CFSE positivity (CDNP^+^ BMN), a situation that did not increase with the addition of LPS (**Figure 5A**). Interestingly, 60% of this CDNP^+^ BMN population expressed CD54, while none of the CDNP^-^ BMN did. Upon LPS addition, the CD54+ phenotype was induced in almost 90% of the CDNP^-^ BMN, increasing to nearly 100% in the CDNP^+^ BMN population (**Figure 5B**). CDNPs were apparently preferentially ingested by CD54+ BMN. We also investigated whether CDNP^+^ BMN and CDNP^-^ BMN differed in their migratory capacity. Remarkably, CDNP^+^ BMN completely failed to migrate in response to CXCL12 (**Figure 5C**). Further phenotypical analysis revealed that 50-60% of the CDNP^+^ BMN expressed CD86 and MHCII, respectively, which were not found in the CDNP^-^ BMN population. Unlike the effect on CD54 expression, LPS did not affect CD86 and MHCII expression (**Figure 5D**). This situation was different for CD80, which was significantly higher (4.6-fold) expressed in the CDNP^+^ BMN without activation. LPS stimulation increased CD80 expression levels in both CDNP^+^ and CDNP^-^ BMN (**Figure 5E**). Next, we assessed the expression levels of the activation marker CD11b in CDNP^+^ and CDNP^-^ BMN. In contrast to the increased expression of CD54, as well as CD80, CD86, and MHCII, there was no difference in CD11b expression between CDNP^+^ and CDNP^-^ BMN (**Figure 5F**). Moreover, the expression of CD11b decreased in the CDNP^+^ BMN upon LPS stimulation. These data demonstrate that CDNPs are selectively ingested by a distinct subset of BMN that exhibits an aged phenotype (CD54+), possesses antigen-presenting features (MHCII+, CD86+), and lacks the ability to migrate in response to CXCL12. Additionally, the expression of CD80 was increased in CDNP^+^ BMN without further stimulation, while the LPS-induced upregulation of CD11b was impaired in CDNP^+^ BMN. Finally, considering the shift towards apoptosis observed in PMA-induced cell death and the complete lack of migration towards CXCL12, we hypothesized that CDNP^+^ BMN might exhibit a higher rate of apoptosis compared to their CDNP^-^ BMN counterparts. Indeed, analysis of active caspase 3 reveals that 76% of the CDNP^+^ BMN undergo apoptosis within 24 hours in culture, which is not found in the CDNP^-^ BMN (**Figures 6A, B**).

In summary, contrary to the current view that intracellular contents act as DAMPs that trigger sterile inflammation, our data indicate that when intracellular content in the form of CDNPs are released into the extracellular space and recognized by innate immune cells, they can induce also anti-inflammatory effects. The recognition of CDNPs by BMN induces a rapid but transient activation, followed by the development of a regulatory and/or apoptotic phenotype. Importantly, this CDNP-induced modulation of BMN reduces further activation by LPS.

## Discussion

Based on previous findings that CDNPs play a regulatory role in inflammation, notably in an autoimmune mouse model for local wound healing and in the systemic CLP model, a condition of severe systemic immune dysregulation, we hypothesized they would have a similar regulatory impact on the function of neutrophils (9, 10). Neutrophils are the main driver cells in both clinical settings (23–25), but the impact of CDNPs on neutrophils under steady state conditions has not been investigated in detail. To find out, we used freshly isolated BMC from healthy mice and cultured them directly without further purification to prevent any kind of pre-activation (26). The exposure of these cultured BMC to CDNPs revealed three major findings: CDNPs (i) induce a transient upregulation and downregulation of CD11b without triggering effector functions; (ii) promote regulatory functions such as secretion of IL-10, a shift toward apoptosis, and upregulation of the antigen-presenting molecule CD80; and (iii) are predominantly ingested by CD54+ non-migrating BMN cells, which undergo apoptosis within 24 hours.

Our first results reveal that CDNPs are readily recognized by resting BMN in culture, as evidenced by the upregulation of CD11b within 45 min, which persisted for 3 hours before ceasing after 24 hours (**Figure 1B**). CD11b, the α-chain of the β2-integrin Mac-1, on the surface of neutrophils, is primarily associated with neutrophil adhesion and migration into tissues. It is also linked with degranulation of secretory vesicles, phagocytosis, and superoxide production (27–29). Despite a slight but statistically insignificant decrease in migration, no significant impact on these classical effector functions was observed in the CDNP-treated groups (**Figures 2A–D**, **3A**, **4E**). It is plausible that the transient increase in CD11b expression results from the activation of danger-sensing receptors that recognize CDNPs as DAMPs. However, this signaling seems insufficient to induce full activation. Interestingly, the addition of LPS to the culture medium suppressed CD11b expression, suggesting that additional stimulation is not the key to triggering effector functions via CDNPs. Instead, CDNPs may shift BMN from an activated state to a hyporesponsive state of tolerance (**Figure 1C**). This indicates that it is not the absence of supplementary signals needed for full activation, but rather that CDNPs potentially induce a state of tolerance in BMN. Another explanation could be the shedding of surface markers due to CDNP-induced apoptosis. However, this seems unlikely, as CDNP^+^ BMN undergoing apoptosis express specific surface markers that are not found on the CDNP^-^ BMN population (**Figures 5B, D, E**).

Secondly, corresponding with the lack of effector functions in the CDNP-treated groups, we observed a significant increase in IL-10 secretion in the BMC (**Figure 2E**) and a proapoptotic effect during the PMA-induced cell death (**Figure 3C**). The secretion of both cytokines was elevated upon exposure to LPS. This increase of IL10 expression following LPS exposure might be explained by the involvement of type I interferons (30). The elevated expression of IL10 in response to CDNP supports the notion that CDNPs induce not only a hyporesponsive but rather a regulatory phenotype of neutrophils that contributes to the resolution of ongoing inflammation. The concept that extracellularly appearing endogenous proteins can have resolving capacities has been previously reported. For instance, it has been reported that HSP27, S100 proteins, or vimentin (31–34) induce IL-10, and annexin A1 has proapoptotic effects (35). MALDI-TOF analysis revealed that CDNPs contain plentiful proteins, including those mentioned above as potential initiators of resolution. These proteins include annexins, S100 proteins, heat shock proteins, calreticulin, and HMGB1 (10) (Data not shown). The question arises whether these individual intracellular proteins would have similar effects when compared to multicomponent particles such as CDNPs. Notably, the most abundant proteins in CDNPs are the members of the Annexin family. Therefore, we specifically investigated whether Annexin A1 and Annexin A5 could individually induce some of the observed CDNP-induced effects on BMN. Culturing BMN with recombinantly produced Annexin A1 and Annexin A5 showed no effect on CD11b expression (**Supplementary Figure 5**), or ROS release (Data not shown). Thus, despite their previously reported anti-inflammatory activities (35–37), Annexin A1 and A5 do not mimic the role of CDNPs in cultured BMN. These data support our hypothesis that intracellular content released upon inflammation or injury will exert its activity rather as multicomponent particles instead of individually soluble molecules. Furthermore, we found that CDNPs affected the antigen-presenting markers CD80, CD86, and MHC II unexpectedly: they selectively upregulated CD80 (**Figure 4A**). Specifically, CDNP-treated neutrophils expressed higher levels of CD80 at early and late time points (**Figure 4A**). In contrast, CD86 and MHC II showed a different expression pattern: the initially low percentages of positive BMN increased after 24 hours of incubation, regardless of CDNP treatment (**Figures 4B–D**). The differing time points of CD80 and CD86 expression are particularly surprising because both molecules are typically upregulated on mature antigen-presenting cells to promote the T cell priming (38). However, distinct expression patterns of CD80 and CD86 have been reported, with CD80 notably associated with immunosuppressive effects (18, 19, 39). Consistent with this sequential expression, one study suggests that CD80 is the initial ligand responsible for maintaining aspects of immune tolerance through interactions with CTLA-4. The subsequent upregulation of CD86 follows as a result of inflammatory stimuli and can override this inhibition, leading to the T cell activation (40). Further research should explore the potential of CDNPs to modulate the interaction between neutrophils and T cells.

Our third finding reveals that CDNPs are selectively ingested by CD54+ BMN (**Figure 5B**) and that these CDNP^+^ BMN, in particular, are more prone to undergo apoptosis while simultaneously expressing antigen-presenting markers (**Figures 5**, **6**). The expression of the adhesion molecule CD54 on neutrophils is primarily associated with their ability to perform reverse transendothelial migration from tissues into the circulation and potentially back to the bone marrow. Our data indicates that CDNP^+^ BMN do not respond to the chemotactic stimulus CXCL12, even though 60% of CDNP^+^ BMN express CD54 under normal conditions, and 90-100% express CD54 following LPS stimulation (**Figures 5B, C**). This lack of migration might be due to the apoptotic phenotype of CDNP^+^ BMN. Furthermore, recent studies have demonstrated that CD54 is associated with phagocytic activity in human and murine neutrophils (17). This raises the question of whether phagocytosis of CDNPs upregulates CD54 or if CD54^+^ BMN preferentially ingest CDNPs. Resolving this question is challenging. On one hand, CD54 expression increases in 20% of BMN over time, with significantly lower expression observed in the CDNP-treated group (**Figure 4D**). On the other hand, CD54 expression is restricted to CDNP^+^ BMN and is strongly upregulated upon LPS stimulation (**Figure 5B**). To further investigate the effect of phagocytosis on CD54 expression, we compared the MFI of CD54 on BMN after ingestion of pHrodo-labeled *E. coli* particles and incubation with CDNPs, LPS, or both (see experimental setup in **Figure 2B**). Interestingly, uptake of *E. coli* particles alone did not affect CD54 expression (**Supplementary Figure 6**). However, LPS stimulation independently increased CD54 expression on BMN, irrespective of *E. coli* particle exposure. These findings indicate that phagocytosis alone does not enhance CD54 expression, suggesting that BMN may already express CD54 prior to CDNP ingestion (**Figure 5B**). Further studies are needed to explore this hypothesis in greater depth. Regarding the effect of CDNPs on CD54 expression, we observed notable variability and no significant changes. Pre-incubation with CDNPs for 45 minutes prior to LPS exposure tended to reduce CD54 expression in both pHrodo− (*E. coli−*) and pHrodo+ (*E. coli*+) BMN, though the reduction was not statistically significant. However, it eliminated the significance between unstimulated and LPS-stimulated pHrodo− (*E. coli−*) and pHrodo+ (*E. coli+*) BMN. These findings suggest that CDNPs might contribute to the modulation of CD54 expression in a context-dependent manner, primarily in BMN that have internalized CDNPs. In addition to its role in phagocytosis, CD54 may also facilitate interactions with T cells during priming by promoting binding to T cells (41, 42). Consistent with the enhanced CD54 expression observed in approximately 20% of BMN after 24 h of culturing, similar trends were noted for CD86 and MHCII (**Figures 4B–D**). The acquisition of antigen-presenting markers and the loss of chemotactic responses in a fraction of neutrophils over time have been previously reported (43). Given the apoptotic phenotype of most of the CDNP^+^ BMN, the question arises: What occurs within a natural microenvironment? Do CDNP^+^ BMN interact directly with T cells before undergoing apoptosis, or are they first phagocytosed by tissue macrophages, which then go on to interact with T cells? Both possibilities are feasible. For example, it has been reported that neutrophils and T cells may encounter each other at sites of inflammation, where they might actively interact (42). One may speculate that upon ingestion, CDNPs are processed, and their proteins are presented as peptides in MHC II to T cells. Another possibility is that neutrophils that ingest intracellular proteins in form of CNDPs become apoptotic and are subsequently phagocytosed by tissue macrophages, which then develop a regulatory phenotype (3, 44). The effects of CDNPs on CD4 T cell differentiation have been demonstrated in murine wound healing and *Leishmania major* infection models, showing a systemic shift from Th1 to Th2 responses (9). However, while it is unlikely, it cannot be ruled out that neutrophils may play a role in antigen presentation. To investigate this further *in vitro*, macrophages, T cells and an inflammatory environment will be required, as the activation of CD4 T cells necessitates co-stimulation or the presence of an adjuvant (41, 42, 45). *In vitro* assays may be limited by the challenge of aligning the optimal timing and microenvironment for all cell types involved. Further, *in vivo* experiments will be needed to investigate whether and how CDNP^+^ neutrophils affect antigen presentation to CD4 T cells. Therefore, the activation and differentiation of CD4 T cells should be assessed by examining the expression of PD-1, Tox, IRF4, and CD44 (46), as well as the emergence of regulatory T cells. Additionally, it would be interesting to analyze the expression of MHC I on CDNP+ neutrophils and its potential effects on CD8 T cells. One of the most suitable *in vivo* models for studying neutrophil function and potential interactions with T cells is the acute respiratory distress syndrome (ARDS), in which a high number of neutrophils accumulate locally in the pulmonary microcirculation (46). In contrast to the CLP sepsis model, which affects the entire peritoneum, the LPS-induced ARDS mouse model allows for a focus on specific tissue niches, such as areas beneath and above the airway epithelium (47). LPS and CDNPs could be injected intratracheally. Additionally, B cell responses might also be influenced by CDNP exposure, given their role as antigen-presenting cells, with or without T cell help. Our lab previously demonstrated that the injection of CDNPs in the *Leishmania major* model shifted *Leishmania major*-specific IgG subclasses from IgG2 to IgG1 (9).

In summary, our data indicate that neutrophil stimulation does not necessarily lead to their complete activation. Instead, it progresses through a multistep process that can be modulated at specific stages. Recent evidence suggests that the tissue microenvironment, particularly specific tissue niches, drives neutrophil heterogeneity and functionality (2). It is plausible that during insults such as infection, immune complex deposition, or tissue injury that provoke neutrophil immigration, each neutrophil is exposed to a distinct tissue microenvironment. Some neutrophils may bind directly to pathogens, immune complexes, or freshly released cell debris via their pattern recognition receptors or Fc gamma receptors, leading to full-scale activation. Other neutrophils might ingest released intracellular content or cell debris that have been modified by exposure to ROS or derive from the release of NETs and develop into a regulatory phenotype. Our data show that CDNPs modulate the expression of surface markers and induce apoptosis in neutrophils. As the response progresses, these modified neutrophils may become predominant, contributing to the resolution of inflammation, potentially by undergoing apoptosis and regulating tissue macrophages through efferocytosis (44, 46). This scenario aligns with prior findings suggesting that extracellular appearing intracellular proteins can serve as both DAMPs and SAMPs (solution-associated molecular patterns), initially triggering an injury-induced response followed by the restoration of homeostasis (48). It has been suggested that neutrophils have the capacity to de-prime back to a basal state after initial priming and may even undergo cycles of priming and de-priming (46). One could speculate that CDNPs might be one of the potential factors supporting this de-priming process before they undergo apoptosis. Given that neutrophils undergo significant changes in cell shape during priming, it will be interesting to investigate the role of CDNPs in the early priming process. One possible approach would be to study the expression of aquaporins (AQP), specifically of AQP9, a molecule typically involved in the regulation of cell size. Assessing the expression of AQP9 could provide new insights (49). Moreover, future studies should aim to precisely define the components within CDNPs that mediate the effects during inflammation and determine whether individual proteins or combinations of several proteins are more effective. Several reports indicate that the therapeutic effects of individual DAMPs, such as HSP10 and HMGB1, were not confirmed in preclinical and early clinical trials (48, 50, 51). This outcome might differ for CDNPs, as they more accurately reflect the *in vivo* situation by consisting of multiple components.

One of the main limitations of this study is that BMN might be immature and not fully functional because they have not yet been released into the bloodstream. To determine whether mature neutrophils recognize CDNPs, we injected CDNPs nine times into the peritoneum of healthy mice. Two hours before harvesting the peritoneal cells, labeled CDNPs were injected. Despite high variability in the percentage of neutrophils migrating into the peritoneum (**Supplementary Figure 7**), we observed that approximately 22% of the neutrophils took up CDNPs and increased CD11b expression *in vivo* (**Supplementary Figures 7A–D**), consistent with the *in vitro* data. These results demonstrate that CDNPs interact with mature neutrophils *in vivo* under steady-state conditions, just as they do with the BMN cultures. Additionally, we previously showed that CDNPs are taken up by neutrophils in the CLP model of sepsis, which improved the disease course by lowering IL-6 levels (10). Furthermore, under inflammatory conditions, such as sepsis, the involvement of immature neutrophils, rather than mature ones, is often observed (52). Another limitation of this study is that we did not investigate the potential underlying mechanisms. For example, to better understand how CDNPs affect apoptosis during the PMA-induced cell death (**Figure 3**), PMA could be replaced by Ionomycin. Ionomycin, a Ca²^+^ ionophore, induces NETosis and cell death more directly and rapidly compared to PMA, which activates protein kinase C in a slower and more sustained process.

Furthermore, unraveling the steps of CDNP-induced apoptosis following ingestion will be crucial to understand to understand their role in potential therapeutic application. It can be speculated that CDNPs, particularly after uptake, interact with the autophagy process. In this context, the specific nature of the proteins present in CDNPs may not play a significant role (53, 54). However, these experiments were designed as a pilot study to provide preliminary insights into the modulatory effects of CDNPs on neutrophils, highlighting the need for further investigation in future studies. Recently, it has been proposed that dying cells do not release intracellular content randomly but in a concerted manner to fine-tune the immune response (55). Based on this hypothesis, CDNPs could be one of these factors, specifically composed to initiate the resolution of inflammation. The structural organization of intracellular molecules in these particles could target multiple signaling pathways in neutrophils (and potentially other immune cells) simultaneously, thereby eliciting a balanced immune response that favors resolution. Further studies will be needed to define how the release of CDNPs shapes local interactions within tissue niches and coordinates neutrophil fate.

## Data Availability

The raw data supporting the conclusions of this article will be made available by the authors, without undue reservation.
