# Supplementary material for: Nanoparticles containing intracellular proteins modulate neutrophil functional and phenotypic heterogeneity
Source: Front Immunol. 2025 Jan 22;15:1494400. doi: 10.3389/fimmu.2024.1494400 (PMC11794831; doi:10.3389/fimmu.2024.1494400)
Supplement: Supplementary file 1 [file DataSheet1.pdf]

Materials provided in the STAR-Method format

Reagent or Resource	Source	Identifier
**Antibodies and Flow cytometry reagents**		
Alexa Fluor 700 Goat anti-Rabbit IgG (H+L) Cross-Adsorbed Secondary Antibody	Thermo Fisher Scientific	A21038
AlexaFluor 488 labeling Kit (Fig. S7)	Thermo Fisher Scientific	A20181
Anti-Histone H3 (citrulline R2 + R8 + R17) Primary Antibody	Abcam	ab5103
Anti-mouse CD16/32 Antibody	BioLegend	101301
Anti-mouse CD16/32 Antibody	Thermo Fisher Scientific	14-0161-82
APC anti-mouse CD86 Antibody	BioLegend	105113
APC Hamster Anti-Mouse CD54	Becton Dickinson	561605
APC Rat Anti-CD11b	Becton Dickinson	561690
APC-Cy7 anti-mouse CD86 Antibody	BioLegend	105029
APC-Cy7 Rat Anti-CD11b	Becton Dickinson	561039
APC anti-mouse Ly-6G Antibody	BioLegend	127613
Brilliant Violet 605™ anti-mouse/human CD11b Antibody	BioLegend	101237
Brilliant Violet 421™ anti-mouse/human CD11b Antibody	BioLegend	101235
brilliant Violet 510™ anti-mouse/human CD11b Antibody (Fig. 6S)	Biolegend	101245
FITC Anti-Myeloperoxidase antibody	Abcam	ab90812
FITC Hamster Anti-Mouse CD54	Becton Dickinson	567898
Fixable Viability Stain 570	Becton Dickinson	564995
Fixable Viability Dye eFluor 780	Thermo Fisher Scientific	65-0865-14
Pacific Blue™ anti-mouse I-Ab Antibody (MHC II)	BioLegend	116421
Pacific Blue™ anti-mouse Ly-6G Antibody	BioLegend	127611
PE anti-mouse CD80 Antibody	BioLegend	104707
PE Hamster Anti-Mouse CD54	Becton Dickinson	553253
PE Rat Anti-Mouse Ly-6G Clone 1A8	Becton Dickinson	551461
PerCP/Cy5.5 anti-mouse Ly-6G Antibody	BioLegend	127615
PE CD11b Monoclonal Antibody	Thermo Fisher Scientific	12-0112-82
mouse FcR blocking reagent (Fig. 6S)	Miltenyi Biotec	130-092-575
Pacific Blue™ anti-mouse CD45 Antibody (Fig. 6S)	Biolegend	103126
PE-Cy™7 Rat Anti-Mouse Ly-6G (Fig. 6S)	Becton Dickinson	560601
BD Horizon™ CFSE	Becton Dickinson	565082
Compensation Beads	Thermo Fisher Scientific	01-2222-41
DHR Dihydrorhodamin123	Sigma Aldrich	D1054
E.coli Opsonizing Reagent	Thermo Fisher Scientific	E2870
E. coli (K-12 strain) BioParticles™, Alexa Fluor™ 488 conjugate	Thermo Fisher Scientific	E13231
pHrodo™ Red *E. coli* Bioparticles™ conjugate	Thermo Fisher Scientific	P35361
**Experimental Kits**		
FITC Active Caspase-3 Apoptosis Kit	Becton Dickinson	550480
Mouse Inflammation, Cytometric Bead Array Kit	Becton Dickinson	552364
PE Active Caspase-3 Apoptosis Kit	Becton Dickinson	550914
Pierce™ Chromogenic Endotoxin Quant Kit	Thermo Fisher Scientific	A39552

**Substances and commercially available solutions**		
0,4% Trypan Blue	Sigma Aldrich (Germany)	T8154
L-Glutamine	Thermo Fisher Scientific	11140050
10X Phosphate Buffered Saline	Thermo Fisher Scientific	AM9625
16% Formaldehyde	Thermo Fisher Scientific	28906
2-Mercaptoethanol	Sigma Aldrich (Germany)	M6250
2-Propanol	Carl Roth (Germany)	CN09.1
4-12% Bis-Tris Midi Protein Gels, 20 well	Thermo Fisher Scientific	WG1402BOX
Acetic Acid	Thermo Fisher Scientific	036289.K3
Acetic Acid	Carl Roth (Germany)	Cat# 64-19-7
Amido black 10 B	Merck (Germany)	1041870025
Ammonium chloride	Sigma Aldrich (Germany)	254134
Bovine Serum Albumin	Thermo Fisher Scientific	B14
Bovine Serum Albumin	Sigma Aldrich (Germany)	A9418
Bromophenol blue	Carl Roth (Germany)	T116.1
Chloroform	Carl Roth (Germany)	3313.2
Coomassie Brilliant Blue	Sigma Aldrich (Germany)	115.444
Crystal Violet	Sigma Aldrich (Germany)	548-62-9
Dimethyl Sulfoxide	Sigma Aldrich (Germany)	67-68-5
Dulbecco’s Modified Eagle’s Medium	Sigma Aldrich (Germany)	D5796
Dulbecco’s Phosphate Buffered Saline, with $MgCl_2$ and $CaCl_2$	Sigma Aldrich (Germany)	D8662
Dulbecco’s Phosphate-Buffered Saline	Corning (USA)	21-031-CV
Dulbecco’s Phosphate-Buffered Saline	Sigma Aldrich (Germany)	D8537
EDTA disodium salt solution	Sigma Aldrich (Germany)	E7889
Ethanol	Decon™ Laboratories, Inc.	2716
Fetal Bovine Serum	Thermo Fisher Scientific	A5256701
Fetal Bovine Serum	Sigma Aldrich (Germany)	F0850
Gentamicin 10mg/mL	Thermo Fisher Scientific	15710064
Hanks’ Balanced Salt Solution, no calcium, no magnesium	Thermo Fisher Scientific	14170120
HEPES	Carl Roth (Germany)	6763.3
HEPES 1M	Sigma Aldrich (Germany)	H0887
Hydrochloric Acid	Thermo Fisher Scientific	L13091.AU
Hydrochloric Acid	Carl Roth (Germany)	4328.3
Immersion Oil	Carl Zeiss (Germany)	10539438
Isopropanol	Th. Geyer GmbH (Germany)	50295857
L-Glutamine 200 mM	Thermo Fisher Scientific	25030081
L-Glutamine 200 mM	Sigma Aldrich (Germany)	G7513
Lipopolysaccharides from Escherichia coli O111:B4	Sigma Aldrich (Germany)	L2630
MEM Non-essential Amino Acid Solution 100X	Sigma Aldrich (Germany)	M7145
Methanol	Carl Roth (Germany)	8388.1
Minimum Essential Medium Eagle	Sigma Aldrich (Germany)	M4526
Natriumhydrogencarbonat	Merck (Germany)	1063290500
NuPAGE Antioxidant	Thermo Fisher Scientific	NP0005
NuPAGE Running buffer	Thermo Fisher Scientific	NP0001
Penicillin-Streptomycin 10 000 U/mL	Sigma Aldrich (Germany)	P4333
Penicillin-Streptomycin 10 000 U/mL	Thermo Fisher Scientific	15140122
Phenylmethylsulfonyl fluoride	Sigma Aldrich (Germany)	10837091001

PMA	Sigma Aldrich (Germany)	P1585
Potassium bicarbonate	Sigma Aldrich (Germany)	237205
Protran™ Nitrocellulose Blotting Membranes: 0.45 µm BA85	Fisher Scientific	09-301-102
Rat serum	Thermo Fisher Scientific (USA)	10710C
RBC lysis buffer	Qiagen (Netherlands)	1067932
Recombinant Mouse CXCL12 (SDF-1α)	BioLegend (USA)	578702
Recombinant Murine TNF-α	Peprotech (USA)	315-01A-20UG
RPMI 1640 Medium	Lonza (Schweiz)	BE15-398F
RPMI 1640 Medium	Sigma Aldrich (Germany)	R6504
SDS Pellets	Carl Roth (Germany)	CN30.1
Sodium azide	Sigma Aldrich (Germany)	822.335
Sodium hydroxide	Carl Roth (Germany)	6771.1
Sodium pyruvate 100 mM	Sigma Aldrich (Germany)	S8636
Sodium Pyruvate 100 mM	Thermo Fisher Scientific (USA)	11360070
Tris hydrochloride	Hoffmann-La Roche (Schweiz)	
Triton^{TM} X-114	Sigma Aldrich (Germany)	X114
Trypsin-EDTA solution	Sigma Aldrich (Germany)	T4049
**Organisms/cell lines/recombinant proteins**		
C57BL6/6J mice	Jackson Laboratories, Bar Harbor, ME (USA)	
C57BL6/6J mice	Charles River (Germany)	
Murine fibroblast-like cell line MC3T3-E1	Deutsche Sammlung von Mikroorganismen und Zellkulturen, Braunschweig (Germany)	ACC210
Embryonale Ferkelnierenzelle-Riebe EFNR (Fig. S7)	Collection of Cell Lines in Veterinary Medicine (CCLV), Friedrich-Loeffner Institut (Germany)	CCLV RIE 86
Recombinant ANXA1	Institute of Biology, University of Lübeck (Germany)	N/A
Recombinant ANXA5	Institute of Biology, University of Lübeck (Germany)	N/A
**Softwares**		
Excel MS Office 365	Microsoft (USA)	N/A
Gen5TM 3.00 Microplate Reader and Imager Software	BioTek Instruments (USA)	N/A
GraphPad Prism 10 ((Version 10.4.0)	GraphPad Software Inc.	N/A
KC Junior (Version 1.41.8)	BioTek Instruments	N/A
Odyssey Infrared Imaging System Application (Version 3.0.16)	LI-COR Biosciences	N/A
AIDA Image Analyzer (Version 3.21.001)	Elysia-Raytest	N/A
FlowJo (Version 10.0.8)	Becton Dickinson	N/A

Attune™ NxT Software (Version 3.1)	Thermo Fisher Scientific	N/A
