# Supplementary material for: Nanoparticles containing intracellular proteins modulate neutrophil functional and phenotypic heterogeneity
Source: Front Immunol. 2025 Jan 22;15:1494400. doi: 10.3389/fimmu.2024.1494400 (PMC11794831; doi:10.3389/fimmu.2024.1494400)
Supplement: Supplementary file 2 [file DataSheet2.pdf]

Supplementary Figure 1

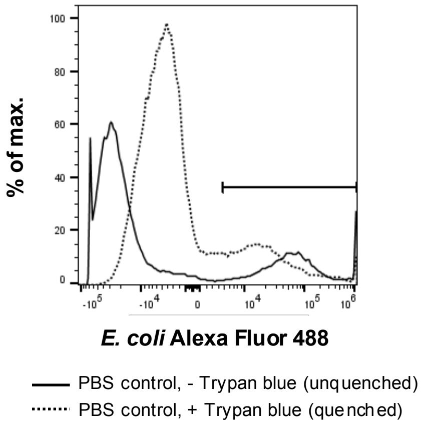

Supplementary Figure 1. Trypan blue diminishes fluorescence of *E. coli* particles only slightly. Opsonized, Alexa Fluor 488-positive *E.coli* particles were added to vehicle-treated BMC and incubated for 15 min at 37°C. To exclude surface-bound *E. coli* samples were measured twice: 1) without and 2) with trypan blue to quench extracellular fluorescence. Quenching resulted in a slightly lower percentage of cells fluorescing at 520 nm.

Supplementary Figure 2

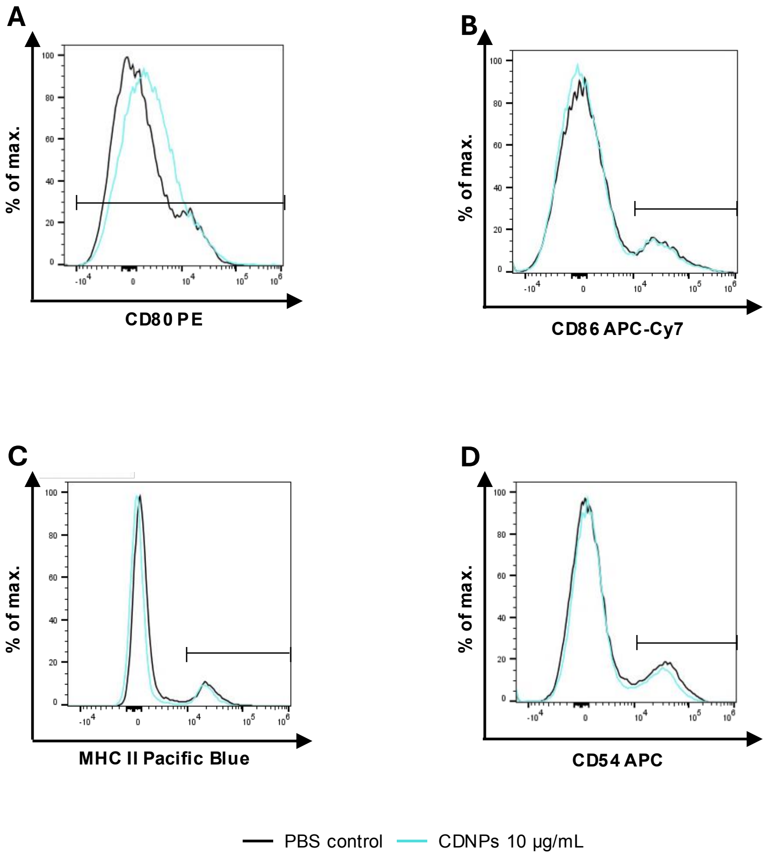

Supplementary Figure 2. Representative histograms for expression of CD80 (A), C86 (B),MHCII (C) and CD54 (D) after treatment with 10µg/ml CDNPs. BMC from BL6 mice were incubated with 10 µg/mL CFSE-labeled CDNPs for 45 min and incubated for further 24 h. BMN were identified as Ly6G+/CD11b+ cells using flow cytometry. Marker expression was determined via the percentage of positive cells (MHC II, CD86, CD54) or the MFI (CD80).

Supplementary Figure 3

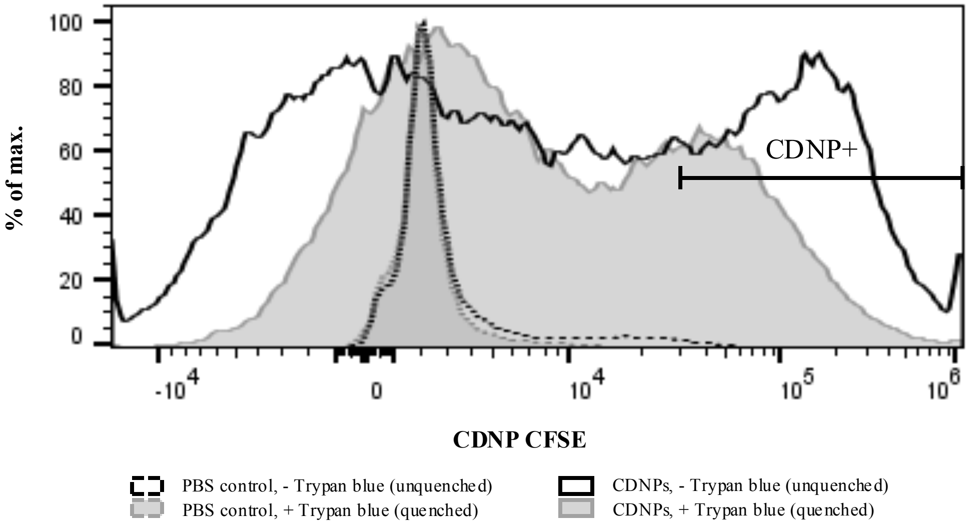

Supplementary Figure 3. Trypan blue alters the fluorescence maximum of CDNP+BMN but does not inhibit it. CDNPs were stained with 10 µM CFSE per 100 µg/mL CDNPs for 20 min at 37°C. Excessive CFSE was quenched with medium containing 10% FCS. Bone marrow cells from BL6 mice were incubated with 10 µg/mL CFSE-labeled CDNPs or CFSE-treated PBS for 24 hours. Samples were measured twice: 1) without and 2) with Trypan blue (0.43 mg/mL) to quench extracellular fluorescence.

Supplementary Figure 4

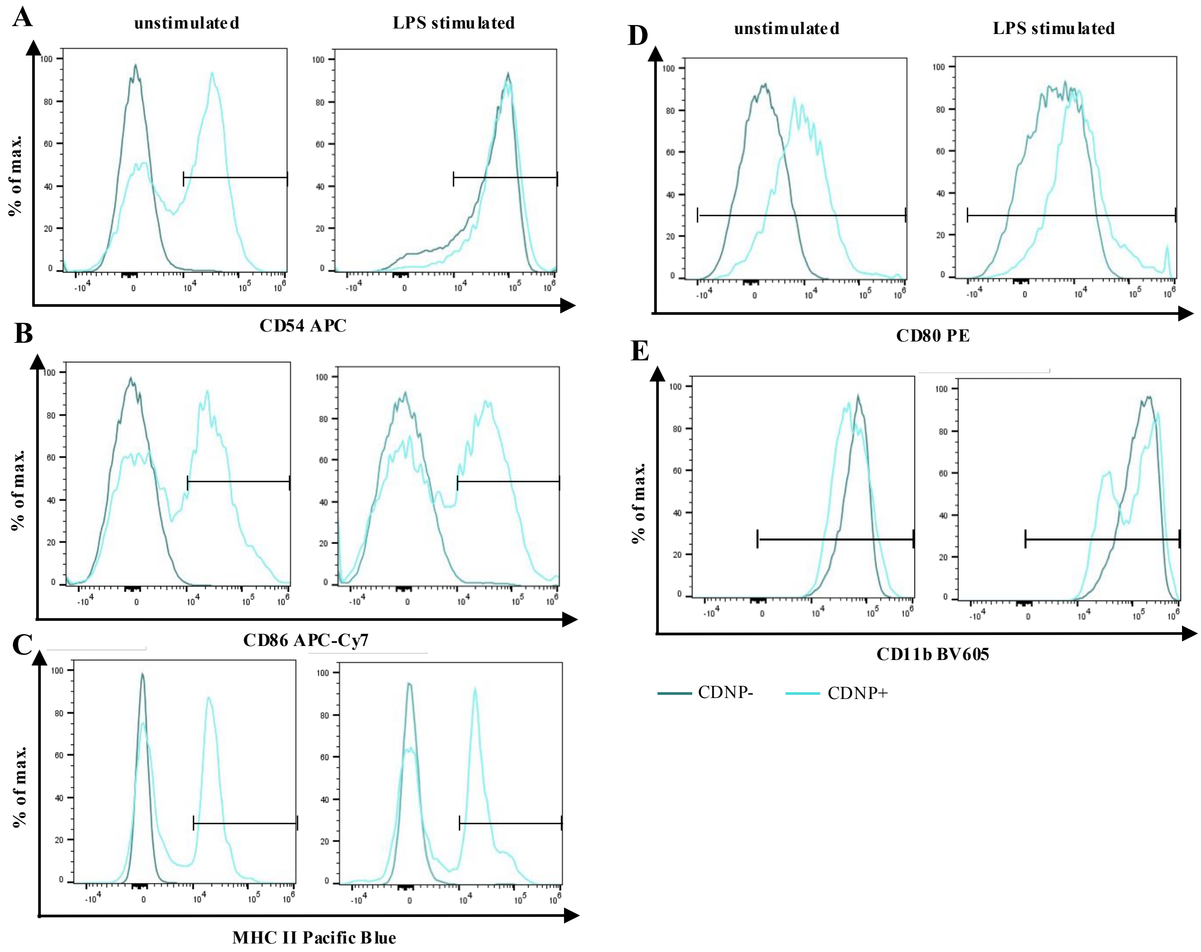

Supplementary Figure 4. Representative histograms for expression of CD54 (A), C86 (B),MHCII (C), CD80 (D) and CD11b (E) for CDNP-BMN and CDNP+BMN.
BMC from BL6 mice were incubated with 10 µg/mL CFSE-labeled CDNPs for 45 min and stimulated with 100 ng/mL LPS for 24 hours. Neutrophils that had ingested CDNP were identified as a CFSE+ subset of Ly6G+CD11b+ cells. Marker expression was determined via the percentage of positive cells (CD54, CD86, MHC II) or the MFI (CD80, CD11b).

Supplementary Figure 5

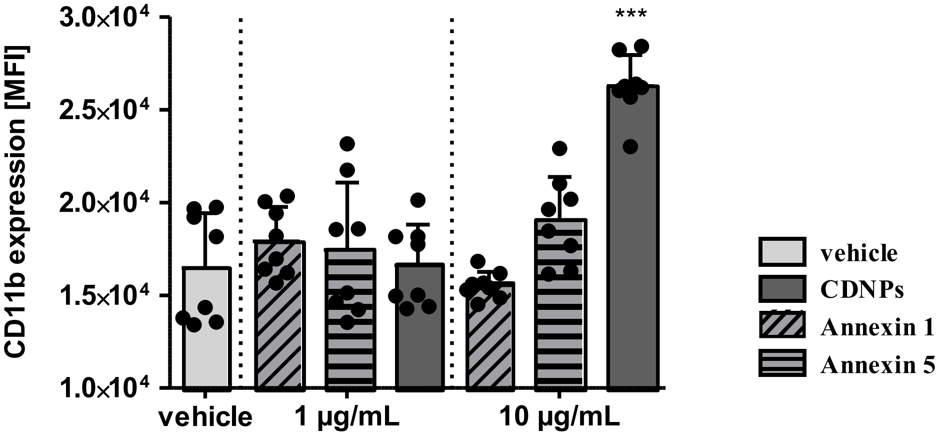

Supplementary Figure 5. Exposure to recombinant murine Annexin 1 and 5 do not affect CD11b expression in BMN. BMC from BL6 mice were incubated with 1 or 10 µg/mL CDNPs, Annexin 1 or Annexin 5 for 45 min. BMN were identified as Ly6G+/CD11b+ cells using flow cytometry. CD11b expression of neutrophils was measured using the MFI of CD11b APC. Data are expressed as mean ± SD, pooled data from 2 independent experiments with n = 8 (4 + 4 pseudo-replicates) per group. Significance was determined using a Two-way ANOVA with Bonferroni posttest, comparing all groups to the PBS control. *** p < 0,001.

Supplementary Figure 6

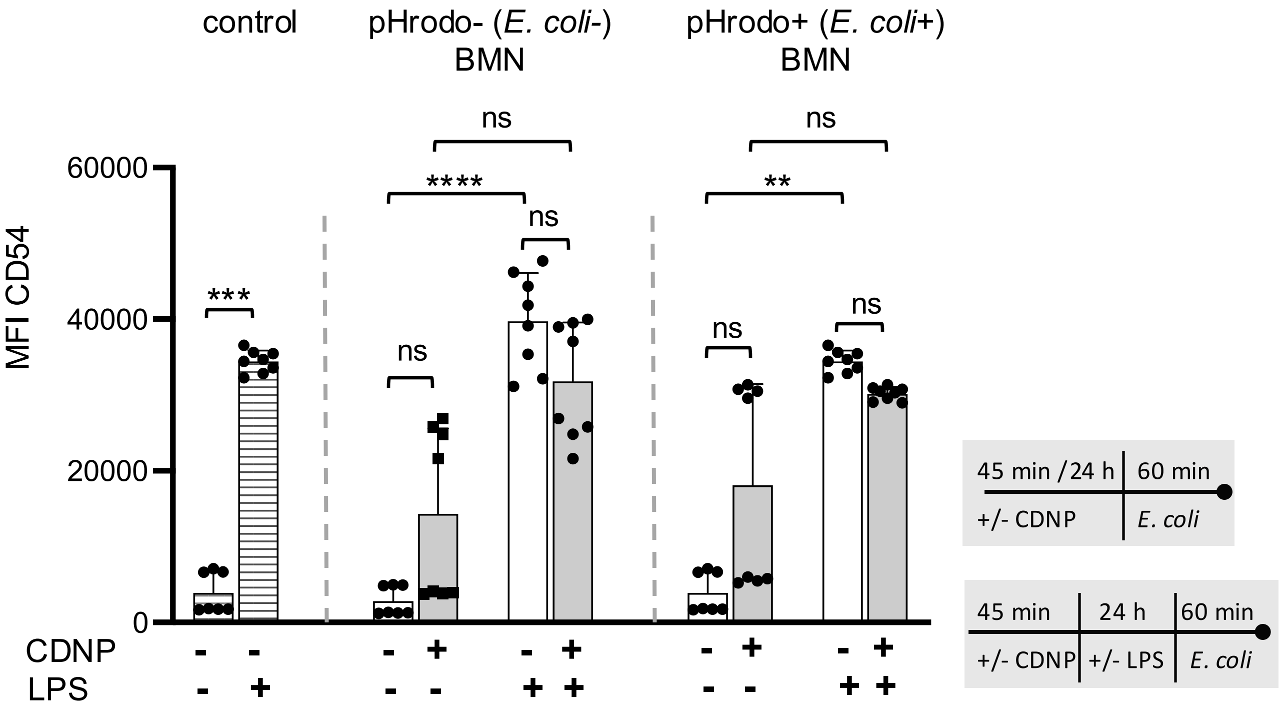

Supplementary Figure 6. Phagocytosis of pHrodo *E. coli bioparticles* does not affect CD54 expression. BMC from C57BL6 mice were preincubated with 10 µg/mL CDNPs for 45 min and subsequently cultured with or without addition of 100 ng/mL LPS for an additional 24 h before exposure to pHrodo™ Red *E. coli* Bioparticles™ conjugates for 60 min. The MFI of CD54 on Ly6G+CD11b+pHrodo+ and Ly6G+CD11b+pHrodo- BMN was determined by flow cytometry. Data are expressed as mean ± SD, pooled data from 2 independent experiments with n = 8 (2 x 4 pseudo-replicates) per group. Significance was determined with the Kruskall-Wallis Test. ** $p < 0.01$ *** $p < 0.001$ **** $p < 0.0001$. Bars: without pHrodo *E. coli* and without CDNPs (striped); with/without pHrodo *E. coli* and without CDNPs (white); with/without pHrodo *E. coli* and with CDNPs (gray). LPS was added as indicated. The incubation periods are illustrated beside the graph.

Supplementary Figure 7

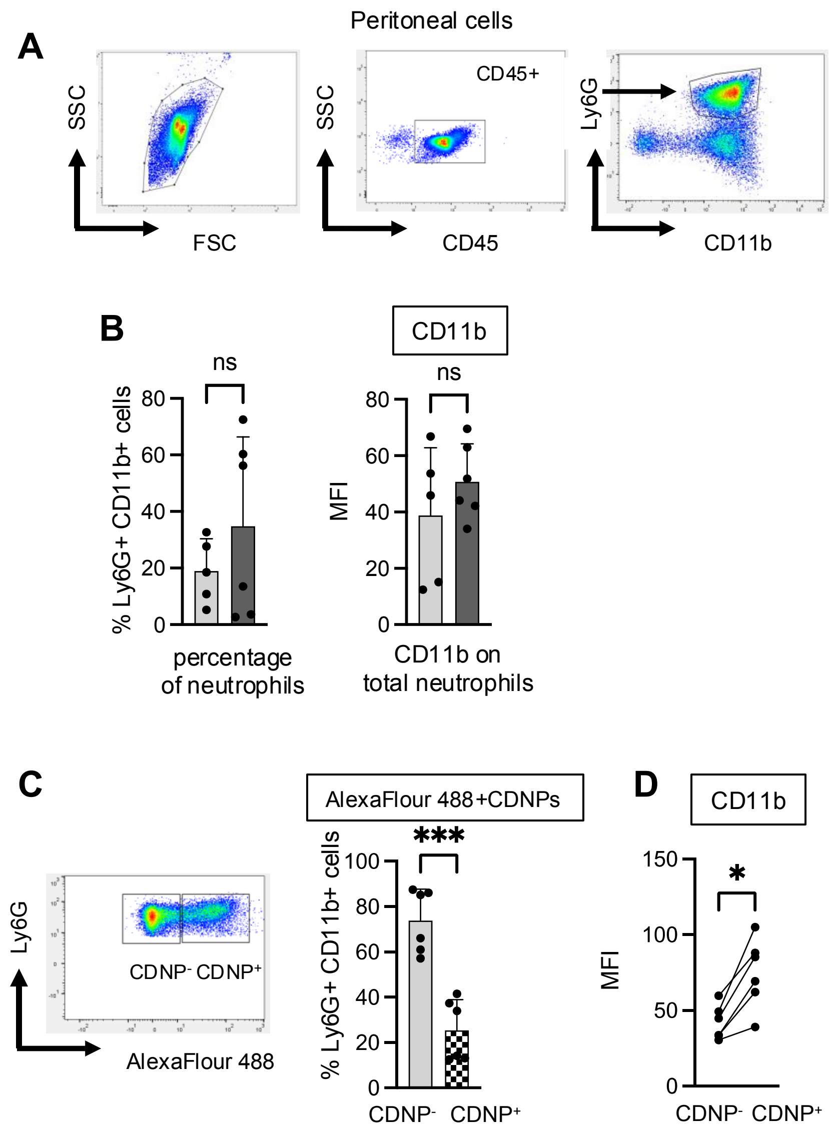

Supplementary Figure 7. CDNP were taken up by peritoneal neutrophils and induced an increase in CD11b expression. 50µg CDNPs (pig derived as described in (2)) were injected 9 times i.p. into BL6 mice*. For the last injection CDNPs were labelled with AlexaFlour 488 and applied 2 h before harvest. Peritoneal washouts were isolated, washed, counted and analyzed. **A)** Neutrophils were identified as Ly6G+CD11b+ cells. **B)** The percentage of neutrophils within the peritoneal lavage (left panel) and the MFI of CD11b on neutrophils in the PBS and CDNP-treated group (right panel) is shown. **C)** The neutrophils that ingested CDNPs were identified by AlexaFlour 488 positivity (left). The percentage of the AlexaFlour 488-labeled subset among the Ly6G+CD11b+ neutrophils was determined by flow cytometry (right). **D)** The MFI of CD11b on neutrophils in the $CDNP^-$ and $CDNP^+$ neutrophils is shown. Data are expressed as mean ± SD, pooled data from 2 independent experiments with n = 2-3 per group. Significance was determined using the Mann Whitney test (B and C) or the Wilcoxon test (D) comparing the PBS and CDNP group *** $p < 0.001$, *$p<0.05$

* The injections of CDNPs were performed between a contact hypersensitivity to TCNB. Please refer to the experimental procedure in the Materials and Methods section for Figure S6. The injection of CDNPs had no impact on ear swelling induced by TCNB.

Material and methods to Supplementary Figure 7

Mice and Experimental approach

8-12 week old female C57BL/6J wild-type (WT) mice were obtained from Charles River Laboratories (Sulzfeld, Germany). The experiment was approved by the local authorities of the Animal Care and Use Committee Kiel, V242-5782/2020 (6-2/20), and performed by certified personnel.

CDNP isolation and preparation

CDNPs were isolated from embryonic pig kidney cells-Riebe (EFNR, Collection of Cell Lines in Veterinary Medicine (CCLV), Friedrich-Loeffner Institute (Germany). They were prepared as described for mouse CDNPs.

Injection of CDNPs

Mice received 200 µl of CDNPs (50 µg protein) intraperitoneally, administered nine times between the first and the second challenge of contact hypersensitivity at the right ear. The contact hypersensitivity reaction was induced as described by (1). PBS or CDNPs were injected at day 5, 6, 7, 12, 13, 14, 17, 18 and 19 after the sensitization and had no effect on the ear swelling reaction. Given that the contact hypersensitivity reaction is highly localized, and sensitization occurred between 6 and 19 days before CDNP injection, we consider it unlikely to have affected neutrophils in the peritoneum.

For the last injection CDNPs were labelled. Therefore, CDNPs were incubated with reactive AlexaFluor 488 (Thermo Fisher Scientific, USA) according to the manufactures protocol. Excessive Dye was removed with provided purification columns. Two hours after the last injection mice were euthanized in an isoflurane saturated glass vessel and killed by cervical location. Peritoneal cells were collected by washing the peritoneal cavity with 2 mL of sterile PBS combined with 5% FBS. The number of cells collected from each animal was estimated using a Neubauer chamber. Cell viability was assessed by trypan blue dye exclusion, and cell populations were defined by flow cytometry. After treating the cells using mouse FcR blocking reagent (Miltenyi Biotec, Germany) for 10 min, cells were stained by incubating the cells with Pacific Blue™ anti-mouse CD45 Antibody (Biolegend, USA) brilliant Violet 510™ anti-mouse/human CD11b Antibody (Clone M1/70, cat number 101245; Biolegend, USA), and PE-Cy™7 Rat Anti-Mouse Ly-6G (Clone 1A8, cat number 560601; Becton Dickinson, USA) for 20 min. Samples were subjected to flow cytometry using MACSQuant® Analyzer 10 (Miltenyi Biotec, Germany).

Timeline for injections of CDNPs

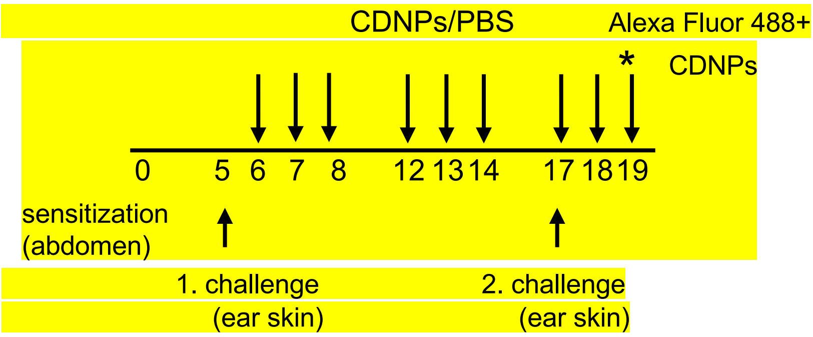

1. El Beidaq A, Link CW, Hofmann K, Frehse B, Hartmann K, Bieber K, et al. In Vivo Expansion of Endogenous Regulatory T Cell Populations Induces Long-Term Suppression of Contact Hypersensitivity. J Immunol. (2016) 197:1567-1576. doi:10.4049/jimmunol.1600508
